# Facilitated Polysulfide Redox Conversion by Delocalized Electrons in MBene Heterointerface for Highly Stable Lithium–Sulfur Batteries

**DOI:** 10.1007/s40820-026-02100-3

**Published:** 2026-02-11

**Authors:** Guifen Wu, Yunmiao Fan, Jiatong Li, Zhaoxi Shen, Yuxiu Xie, Peixun Yang, Jun Pu

**Affiliations:** 1 Key Laboratory of Functional Molecular Solids (Ministry of Education), Anhui Provincial Engineering Laboratory for New-Energy Vehicle Battery Energy-Storage Materials, College of Chemistry and Materials Science, Anhui Normal University, Wuhu, 241002 People’s Republic of China; 2 College of Chemistry and Materials Science, Key Laboratory of Analytical Science and Technology of Hebei Province, Institute of Life Science and Green Development, Hebei University, Baoding, 071002 People’s Republic of China; 3 State Key Laboratory of Multiphase Complex Systems, Institute of Process Engineering, Chinese Academy of Sciences, Beijing, 100190 People’s Republic of China

**Keywords:** 2D MBene heterointerface, Electron delocalization, In situ XAFS and XANES, Polysulfide conversion, Bifunctional catalysts

## Abstract

The shuttle effect of lithium polysulfides (LiPSs) and sluggish redox kinetics severely restrict the development of high-energy lithium–sulfur (Li–S) batteries. To alleviate this issue, this study adopts an in situ design strategy to construct tungsten carbide (WC) nanocrystals on the surface of two-dimensional (2D) tungsten boride (WB)-based MBene, creatively forming a WB@WC heterostructure to optimize the adsorption–migration–catalysis mechanism of LiPSs. The WB–WC heterointerface reduces the reaction energy barrier of LiPSs due to the electron delocalization effect and promotes the deposition/dissociation of Li_2_S and the transfer of charge. In situ Raman verified that WB@WC can effectively inhibit LiPSs shuttling. In situ X-ray absorption fine structure spectroscopy (XAFS) characterizations further explored the dynamic change of W valence state during LiPSs redox cycle. Encouragingly, the WB@WC-modified Li–S cell delivers an initial capacity of 1277 mAh g^−1^ at 0.2 C. It exhibits extremely stable cycling performance at 2 C, with a low-capacity decay rate of only ~ 0.024% per cycle. Even under sulfur loading of 7.92 mg cm^−2^, high capacity of 7.9 mAh cm^−2^ can still be achieved. This work provides an effective method for regulating the activity of MBene-based catalysts.

**Supplementary Information:**

The online version contains supplementary material available at 10.1007/s40820-026-02100-3.

## Highlights

- A multifunctional 2D tungsten boride (WB)@tungsten carbide (WC) heterostructure was innovatively fabricated by a fluorine-free MBene etching process and in situ carbonization technology.
- The enhanced local electron delocalization effect at the heterointerface effectively suppressed the shuttle effect and improved the reaction kinetics, achieving an area capacity of up to 7.9 mAh cm^−2^ and a capacity attenuation as low as ~ 0.024% per cycle.
- In situ X-ray absorption spectroscopy verified the catalytic mechanism of WB-based MBene.

**Supplementary Information:**

The online version contains supplementary material available at 10.1007/s40820-026-02100-3.

## Graphical abstract

**Figure:**
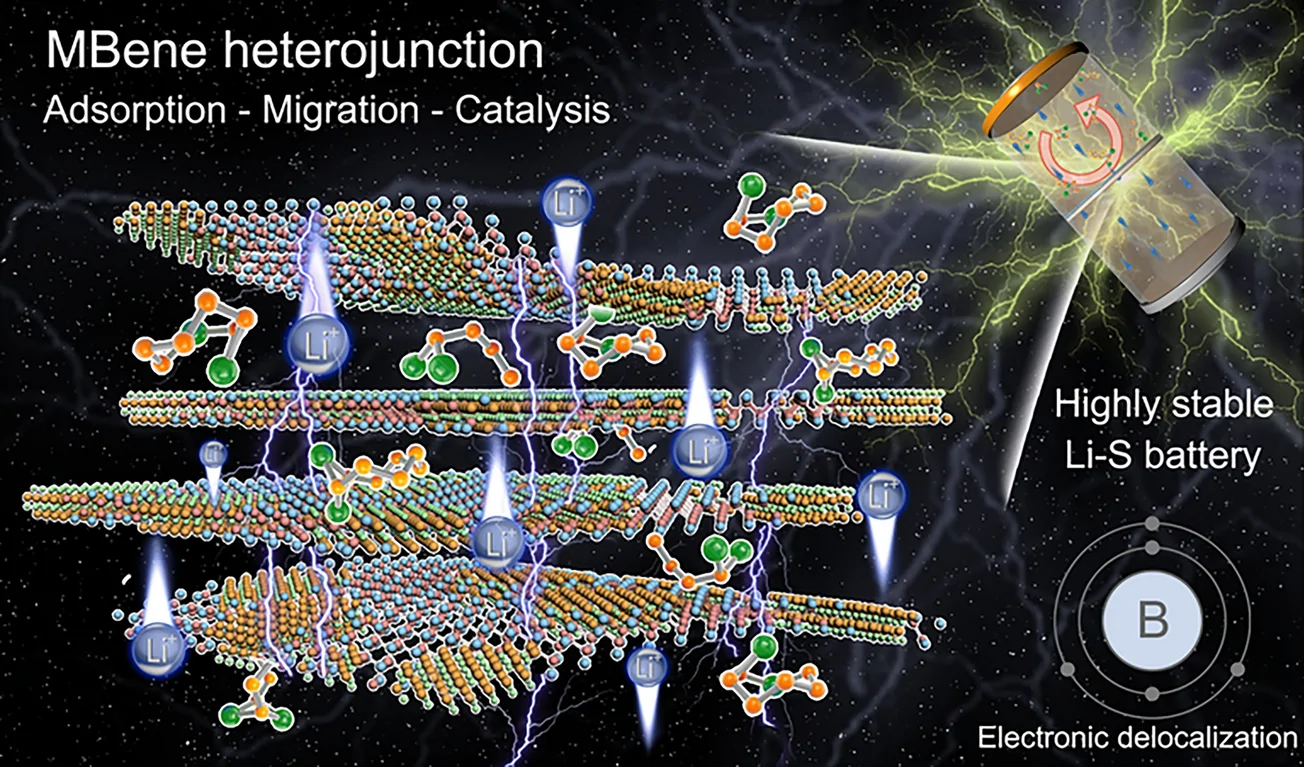

## Introduction

Lithium–sulfur (Li–S) batteries are widely recognized as one of the core candidates for next-generation high-energy storage systems, thanks to their ultra-high theoretical energy density of 2600 Wh kg^−1^ and the characteristics of low cost and environmental friendliness [1, 2]. However, the its commercialization is still constrained by three core challenges. On the one hand, soluble lithium polysulfide (LiPSs, Li_2_S_*n*_, *n* = 4–8) dissolves and shuttles in the electrolyte, leading to the loss of active substances, a decline in Coulombic efficiency, and a deterioration in cycle stability. On the other hand, the insulating property of sulfur with the discharge products (Li_2_S_2_/Li_2_S) hinders the transfer of charge [3–5].

In recent years, efficient LiPS adsorption and catalytic materials have been introduced into Li–S batteries as cathode hosts or separator modified layers to address these issues. [6–8]. Two-dimensional (2D) transition metal (M) compounds (such as MXene, MoS_2_, etc.) are widely used due to the unique layered structures and rich surface chemical properties [9–12]. Among them, the novel MBene (2D transition metal borides) has attracted much attention. From a structural perspective, this material is essentially a B-based derivative formed by the substitution of C/N atoms in MXene with B atoms [13, 14]. On the one hand, because the electronegativity of B (2.04) is much lower than that of C/N, it has a weak attraction to electrons of metal atoms, resulting in a high density of electron clouds on metal surface. In addition, the empty p orbital of B can form a strong coordination bond (B–S) with the lone pair of electrons in the S atom of LiPSs. The synergy of the two significantly enhances the adsorption energy of MBene for LiPSs [15, 16]. On the other hand, the bond energy between B atoms and transition metals is lower than the M–C bond energy of MXene, which can enhance the electron freedom and transfer rate of transition metal atoms. Moreover, the low electronegativity of boron atoms makes the M–B bond more chemically inert and less likely to undergo side reactions with Li-ions and electrolytes. The low valence electrons of B result in strong electron delocalization within the MBene layer, and the intrinsic conductivity reaches about 10^5^ to 10^6^ S m^−1^ [17–19]. Among all the MBene materials, the number of unpaired electrons in the d orbitals of the W atomic nucleus is relatively large, indicating a significant catalytic potential. Meanwhile, as a typical MBene, tungsten boride (WB) shows excellent chemical stability and electron transport capacity [20, 21]. However, a single WB material still has the problem of insufficient catalytic activity and is difficult to efficiently promote the deposition/dissociation process of Li_2_S simultaneously. Furthermore, the specific mechanism by which MBene-based materials act as catalytic materials to accelerate the kinetics remains unclear.

To solve above problems, in situ construction of heterointerface on WB-based MBene is regarded as a highly promising strategy. This in situ heterointerface, while inheriting the advantages of each component, can generate an internal electric field, further promoting the “adsorption–migration–catalysis” synergistic effect of active sulfur species [22]. For example, Zhang et al*.* achieved efficient anchoring and rapid conversion of LiPSs by redistributing the interface charges of V_2_O_3_/V_8_C_7_ heterostructures [23]. Yao et al*.* carried out in situ nitriding on NiO nanoparticles and prepared a defect-rich NiO-Ni_3_N heterogeneous material [24]. The abundant surface area and defects provided LiPSs with sufficient adsorption–catalytic sites. Similarly, the in situ construction of MBene heterostructures is expected to further enhance the efficiency of LiPSs reactions. However, the current WB-based MBene phase derivatization strategies mostly focus on the etching preparation of single-phase borides, and the research on the in situ direct heterostructure construction is still blank. Although the Mo-based MBene heterostructure design has been confirmed, the ex situ stacking process may have the defect of loose bonding at the heterointerface. Based on the excellent corrosion resistance and unique electronic structure of WB, the construction of multi-component heterostructures through in situ doping and phase transition is expected to optimize the charge transport efficiency and interface stability, paving new avenues for applications in energy storage, catalysis [22]. Therefore, how to precisely control the WB components in the heterostructures to achieve performance optimization still requires systematic exploration.

**Fig. 1:**
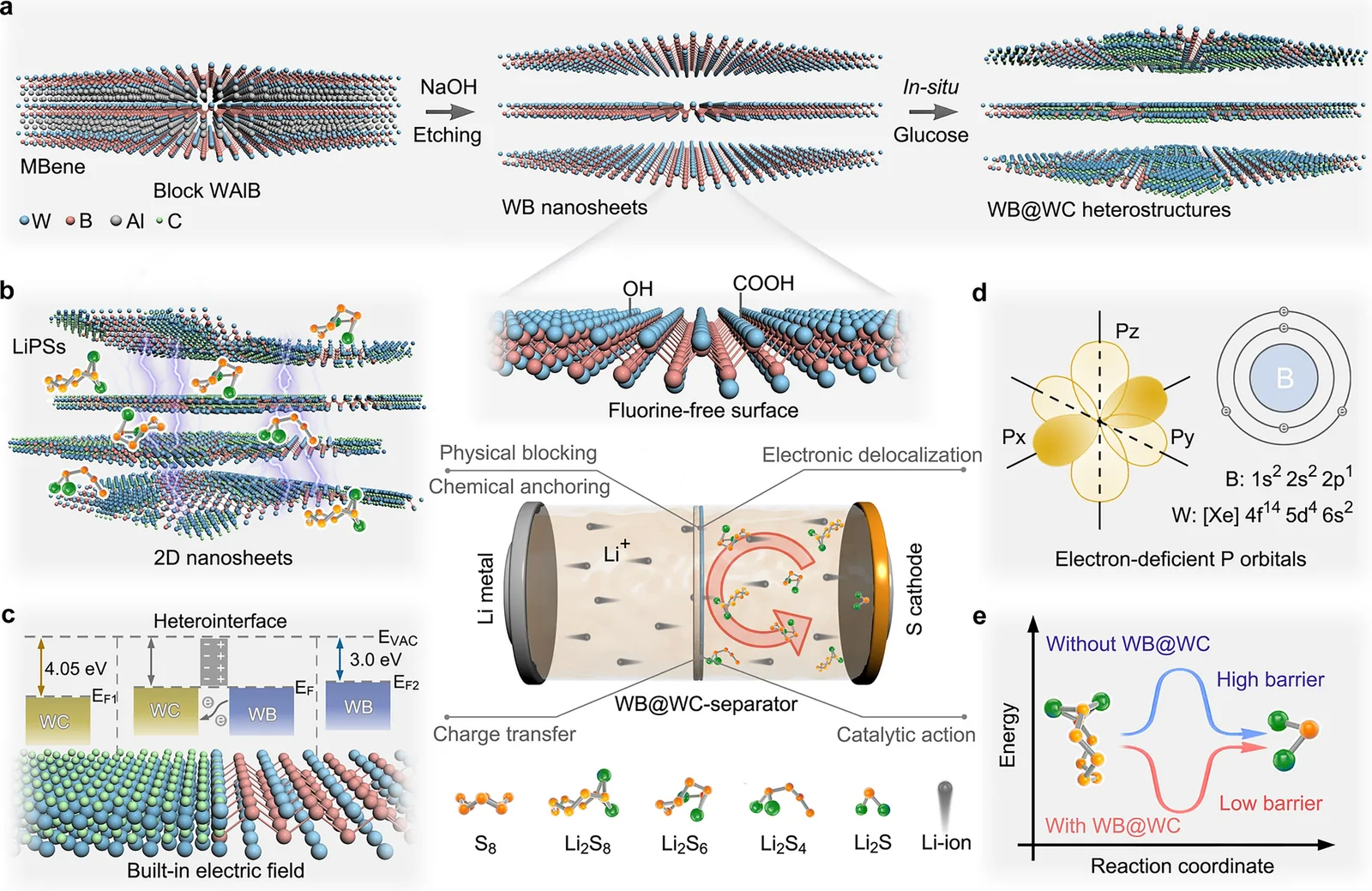
Mechanism schematic diagram. **a** Flowchart of fluorine-free preparation of in situ WB@WC heterostructure nanosheets. **b** Physical obstruction and chemical adsorption. **c** Built-in electric field effect at heterointerfaces. **d** Electron delocalization effect. **e** Energy barrier mechanism of 2D WB@WC in the Li–S system

Herein, this study innovatively proposes a transformation path of “MBene → in situ carburization → boride–carbide heterostructures” (Fig. 1a). Using the WAlB phase as the starting material, the WB 2D structure was obtained by removing the Al layer through F-free etching. Then, an in situ carbonization strategy was adopted to controllably grow tungsten carbide (WC) nanocrystals on the surface of WB and construct WB@WC heterostructures as a modification coating for Li–S separators. The 2D configuration with high specific surface area not only prevented the diffusion of LiPSs through physical confinement, but also chemically anchored LiPSs by providing sufficient sites (Fig. 1b). Moreover, the lattice mismatch between WB and WC induced more defect sites, thereby increasing the density of catalytic active centers [25]. The built-in electric field formed at the heterointerface of WB and WC effectively enhanced the charge transfer abilities of LiPSs (Fig. 1c) [1]. The B element with electron deficiency (empty p orbitals) promoted the redox process of LiPSs through enhanced electron delocalization (Fig. 1d). Experimental characterization and theoretical calculation systematically verified the reduction in the reaction energy barrier of sulfur species by this heterogeneous pairing (Fig. 1e). In situ Raman and in situ X-ray absorption fine structure spectroscopy (XAFS) techniques, respectively, revealed the inhibitory effect of WB@WC on the shuttle effect and the dynamic valence change of W catalytic active center on the electrochemical reaction. As a result, the initial capacity of the Li–S cell with WB@WC-modified separator was up to 1277 mAh g^−1^ at 0.2 C, and the rate capacity was 538 mAh g^−1^ at 4 C, while it exhibited an extremely low-capacity decay rate of ~ 0.024% per cycle. Even under a high sulfur loading of 7.92 mg cm^−2^, the cathode based on this heterostructure still demonstrated an area capacity of 7.9 mAh cm^−2^ and remarkable stability. Therefore, the construction of the WB@WC heterointerface engineering provided inspiration for the development of Li–S batteries with high rate and long life. More importantly, this strategy breaks through the limitation of the single-phase structure of MBene derivatives, achieving the regulation from single-phase borides to boride–carbide heterostructures, and providing a brand-new idea for the multifunctional design of MBene.

## Experimental Section

### Preparation of WB@WC Heterostructure

First, 0.1 g of WAlB powder was added to 20 mL NaOH solution (1 wt%), and the mixture was stirred continuously with a magnetic stirrer at room temperature for 24 h. The corresponding reaction product was rinsed repeatedly with deionized water and ethanol alternately until the rinsing solution became neutral. Subsequently, the product was dried at 60 °C for 12 h, and finally the precursor was obtained. Next, 0.1 g of dried precursor was dispersed in deionized water. About 0.54 g of glucose was added to the dispersion, and the mixture was stirred continuously for 30 min. Then, the mixture was transferred to a reaction kettle and reacted at 150 °C for 1 h. Finally, the product was centrifuged, washed, and dried in sequence to get WB@WC. Both the comparison samples WC and WB were made of commercial nanomaterials.

### Preparation of Modified Separator

Mixtures of 70 wt% WB@WC, 20 wt% CNTs, and 10 wt% polyvinylidene fluoride (PVDF) binders were, respectively, dissolved in N-methyl-2-pyrrolidone (NMP), and then coated on Celgard 2400 PP separators.

### Materials Characterization

The morphology and microstructure of the materials were observed by scanning electron microscopy (SEM) (Hitachi Regulus 8100), transmission electron microscopy (TEM) and high-resolution TEM (HRTEM) (FEI Talos F200x, 200 kV). The crystal phase was characterized by X-ray powder diffraction (XRD) (Rigaku Smart Lab). The high-angle annular detector dark-field (HAADF) based scanning transmission electron microscopy (STEM), and X-ray spectroscopy (EDS) were also characterized on above Talos F200x device. The atomic valence states were analyzed using X-ray photoelectron spectroscopic (XPS) (Thermo Scientific ESCALAB). The ex situ or in situ element composition and electronic state were obtained using XAFS, wavelet transformation-based XAFS (WT-XAFS) and X-ray absorption near-edge structure spectroscopy (XANES) (easyXAFS300 +). Raman spectroscopy tests were conducted by a Princeton Instruments SP-2500 spectrometer with a 488 nm laser excitation. For in situ cell, a Be window was selected as the observation window, and a 0.6 cm × 0.6 cm observation hole was cut at the center of the metal Li anode. The work function analysis was using ultraviolet photoelectron spectroscopy (UPS). The valence band (VB) spectra were measured with a monochromatic He I light source (21.2 eV) and a VG Scienta R4000 analyzer. A sample bias of − 5 V was applied to observe the secondary electron cutoff (SEC). The work function (Φ) can be determined by the difference between the photon energy and the binding energy of the secondary cutoff edge.

### Visual Polysulfide Adsorption and Permeation Tests

Sublimated sulfur powder and Li_2_S were added to the mixed solvent of 1,3-dioxopentane (DOL) and 1,2-dimethoxyethane (DME) (v/v, 1/1) in a ratio of 3:1. After heating and dissolving, a Li_2_S_4_ solution with a concentration of 2.7 mmol L^−1^ was obtained. The same volume of DOL/DME-based electrolyte was added to both left and right chambers of the H-type electrolytic cell for a permeation test, with the left chamber containing Li_2_S_4_ at the above concentration. Modified PP and blank PP separators were, respectively, used for isolation. Note that both the visual adsorption and penetration tests were conducted in a glove box filled with Ar gas. For visual adsorption, the brownish-yellow Li_2_S_6_ solution was prepared using above method. Equal masses of W-based materials and CNT were separately added to the solution.

### Battery Assembly and Electrochemical Measurements

The complex sulfur (prepared by CNTs and elemental sulfur in a mass ratio of 3:7 at 155 °C), acetylene black and PVDF were mixed in a mass ratio of 8:1:1 and coated on the Al foil. After drying, the Al foil was cut into discs with a diameter of 12 mm, and its sulfur content was approximately 1.1–7.9 mg cm^−2^. The prepared sulfur cathode, WB@WC-modified separator or PP separator, Li anode, and DOL/DME electrolyte containing 1 mol L^−1^ bis(trifluoromethane)sulfonamide lithium (LiTFSI) and 0.2 mol L^−1^ LiNO_3_ additive were assembled into CR2032 cells. The ratio of electrolyte to sulfur (E/S) was approximately 15 μL mg^−1^ under normal loading and approximately 7.5 μL mg^−1^ under high loading. The charge–discharge, cycle stability, and galvanostatic intermittent titration technique (GITT) tests were conducted using the LAND battery test system. Cyclic voltammetry (CV) and electrochemical impedance spectroscopy (EIS) (10 mHz–100 kHz) were tested using the CHI660E electrochemical workstation.

For symmetrical cells, the active substance, CNT, and PVDF were ground in NMP at an 8:1:1 mass ratio, then evenly coated onto Al foil. After drying, two identical electrodes were assembled with different separators, respectively, to form symmetrical cell. By mixing sulfur with Li_2_S in a molar ratio of 5:1, a 0.5 mol L^−1^ Li_2_S_6_ electrolyte based on LiTFSI/DOL/DME was prepared. The voltage range for CV testing was − 0.8 to 0.8 V, and the sweep rate was 50 mV s^−1^.

## Results and Discussion

### Heterostructure Design and Phase Characterizations

**Fig. 2:**
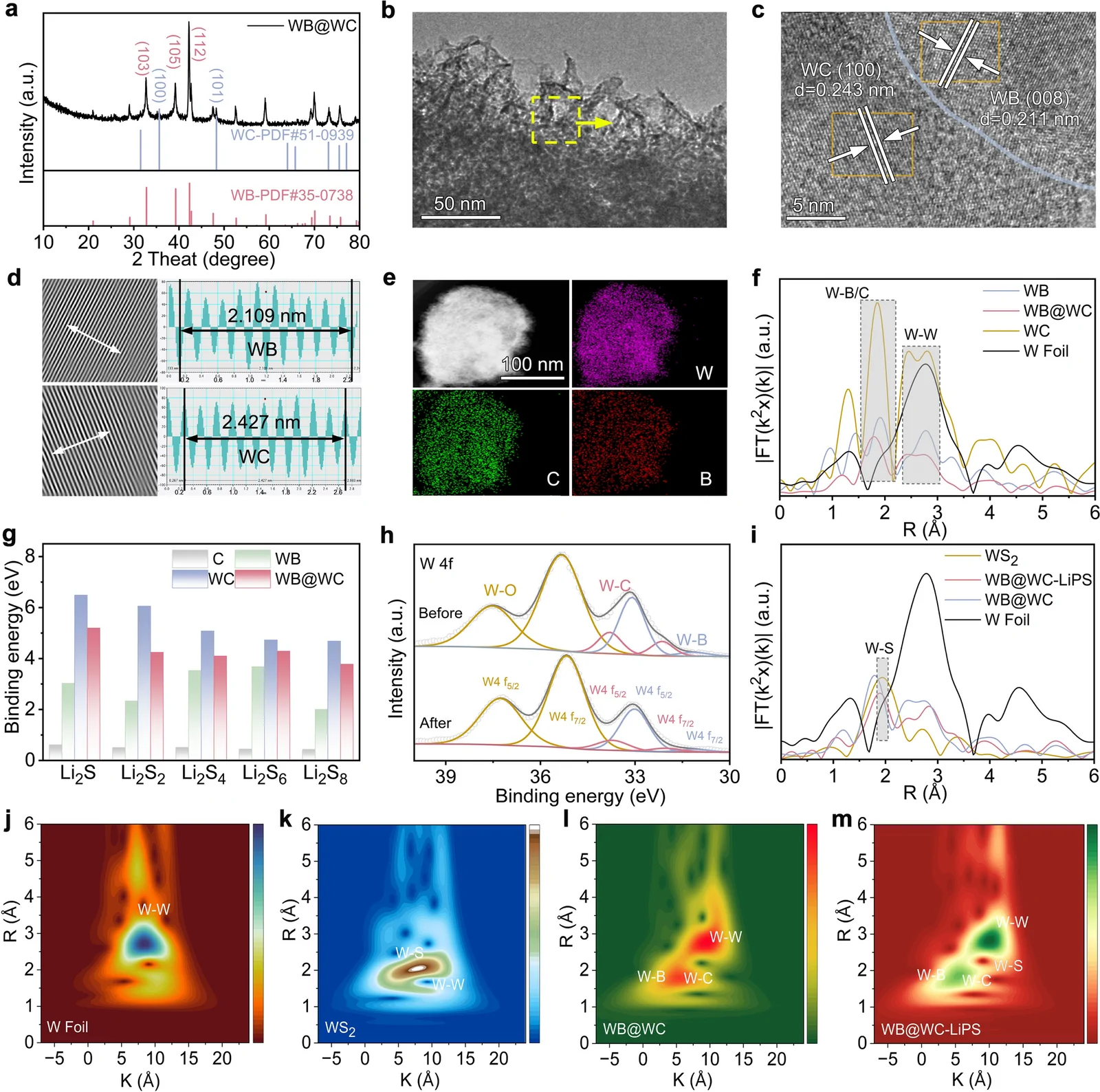
Phase characterization. **a** XRD pattern of WB@WC. **b–d** TEM, HRTEM, lattice spacing, and corresponding Fast Fourier Transform (FFT) of WB@WC nanosheets. **e** HAADF-STEM and areal elemental mapping. **f** W L-edge extended XAFS spectra of WB, WB@WC, WC, and W foil. **g** Calculated binding energies of different sulfur species on multiple matrices. **h** High resolution XPS spectra of W 4*f* before and after Li_2_S_6_ adsorption. **i** XAFS of WB@WC sample after adsorbing LiPSs, as well as other related samples. **j–m** WT-XAFS of different W-based samples

Figure 1a shows the process of preparing WB@WC. Unlike the common etching techniques of HF and its derivatives for MXene. The F-free MBene surface showed no strongly reactive functional groups, which enabled it to achieve superior stability in ether electrolytes [26, 27]. In addition, this process effectively avoided the residual surface toxicity of HF and the excess F-ions [26, 28]. The former will damage the electrode components. Although an appropriate content of the latter is beneficial to the stability of Li anodes, the electronic and ionic conductivities of LiF formed by excessive F-ions are only 10^−10^ and 10^−31^ S cm^−1^, respectively [29]. Given that sulfur cathodes are inherently electrically insulating, excessive LiF generation leads to an increase in battery resistance, which further impairs charge transfer kinetics. During the in situ carburizing process, the glucose-derived carbon source reacted with the surface of WB, and WC nanocrystals grew in situ to form a tightly bonded with WB. This effectively addresses the critical challenge of interfacial bonding instability in heterostructure fabrication [22]. Different with the XRD pattern of the original MBene (Fig. S1), no characteristic peak of Al element was observed in WB sample (Fig. 2a), which indicated that the Al element in WAlB has been completely removed [30, 31]. The diffraction peaks after in situ carbonization corresponded, respectively, to the standard cards of WB and WC. The peaks of 32.6°, 39.2°, and 42.2°, respectively, belonged to the WB crystal planes (103), (105), and (112), while the peaks of 35.6° and 48.3°, respectively, belonged to the WC crystal planes (100) and (101), demonstrating the successful synthesis of heterogeneous materials. Furthermore, by regulating the temperature gradient (100, 150, and 180 °C), samples with increasing carburization degrees (WC mass fraction 4%, 10%, and 13%) were obtained. The degree of carburization was initially regulated. By comparing the XRD patterns of different products (Fig. S2), the characteristic diffraction peaks of WB were clearly present at all temperatures, and the peak shapes did not broaden or show significant intensity attenuation, indicating that the in situ growth of WC did not damage the overall framework of the WB crystal. TEM observed that the synthesized WB@WC presented an ultrathin 2D form (Fig. 2b), which was significantly different from the massive structure of the WAlB precursor (Fig. S3). HRTEM images in Fig. 2c, d reveal lattice fringes of the two components, with a lattice spacing of ~ 0.211 nm corresponding to the (008) crystal plane of WB and a lattice spacing of ~ 0.243 nm belonging to the (100) crystal plane of WC. Furthermore, the energy dispersive EDS mapping results are shown in Figs. 2e, S4, and S5. The W, B, and C elements in the nanosheets were uniformly distributed, further indicating the successful construction of the WB@WC heterostructures. Based on the different work functions of WB and WC (Fig. S6), their heterogeneous interfaces can form an effective built-in electric field, which is conducive to the diffusion of charges [1].

XAFS was adopted to analyze the coordination around W atoms (the corresponding fitting details are shown in Fig. S7 and Table S1). As shown in Figs. 2f and S8, the XAFS curve of WB@WC in R space exhibited two characteristic peaks: the main one was at approximately 1.81 Å, and the sub-strong one was at about 2.73 Å. Combined with comparative analysis with W foil, WB, and WC, these two characteristic peaks corresponded to the W–B/C bond and W–W bond in the first coordination shell, respectively [21, 32]. The intensities of each peak and the R values reflected the coordination environment of W with B and C atoms in the original state of the material, confirming the existence of W–B and W–C coordination.

### Mechanism Exploration and Verification

The above phase characterization fully demonstrates the formation of the heterogeneous interface between WB and WC. The electron delocalization at the heterointerface of inorganic materials would play a core role in the regulation of LiPSs reactions [6, 12]. Electron Localization Function (ELF) analysis in Fig. S9 shows that electron delocalization and aggregation behaviors occurred at the WB@WC heterointerface. This result not only confirms the rearrangement of electron density but also uncovers the delocalized distribution of d-orbital electrons [1]. On the one hand, WC itself is a typical metallic conductor (the p-orbital of the C atom and the d-orbital of the W atom form a conductive energy band). It can endow the heterostructure as a whole with excellent electrical conductivity, preventing the obstruction of electron transmission caused by the sole existence of WB. On the other hand, the electronegativity of C atoms is between that of W and B. After WB–WC interface formation, W atoms on both sides exhibit different electron densities due to the distinct chemical environments. This difference drives electron migration through a weak electronic induction effect, eventually forming a delocalized electron system across the WB and WC phases. From the perspective of micro-mechanism, the d-orbital of W, empty p-orbital of B, and p-orbital of C undergo effective overlap at the interface. This overlapping region forms a channel for electron transport across the interface, breaking the confinement of electrons within a single phase and enabling cross-phase delocalization. When LiPSs are adsorbed at the interface, delocalized electrons can quickly transfer to LiPSs molecules, significantly reducing the energy barrier for redox conversion [5].

Figure 2g shows the binding energies of different substrates for active sulfur species calculated based on density functional theory (DFT). Compared with WC and WB, WB@WC showed a suitable adsorption energy of active sulfur species. Excessive binding might lead to the destruction of the sulfur species structure [33]. However, its value was higher than that of single-component WB. This improvement in adsorption capacity might stem from the enhancement of the d-band center (Fig. S10). After equal masses of WB, WC, and WB@WC were, respectively, immersed in Li_2_S_6_ solution (~ 2.7 mmol L^–1^), the single-component WC and WB systems, respectively, presented different degrees of light yellow, while the WB@WC group solution was nearly colorless (Fig. S11). It should be noted that this adsorption phenomenon was not contradictory to the theory. The core reason was the difference in effective specific surface area caused by the material morphology. WB@WC was a lamellar structure, while WC was a commercial block sample (Fig. S12). Under the condition of equal mass, the former exhibited more exposed active sites and a higher actual adsorption capacity for LiPSs, so the solution decolorization was more obvious. The blocky morphology of the latter leaded to a small number of effective adsorption sites. Although its intrinsic adsorption energy was high, the actual adsorption capacity was limited, so the solution remained light yellow.

To further verify its interaction with LiPSs, XPS and XAFS were performed to explore the changes in the atomic coordination environment and chemical state of WB@WC before and after it adsorbed Li_2_S_6_. As shown in Fig. 2h, the W 4*f* orbital split into multiple peaks of low binding energy and high binding energy due to spin–orbit coupling, which were assigned to the W–B, W–C, and W–O bonds [20, 34]. The W–O peaks were caused by inevitable surface oxidation [34]. Similar to previous literatures, W 4*f* peaks shifted to lower binding energies after adsorption, indicating that W gained electrons from LiPSs, which might be related to the possible formation of new chemical bonds between W and polysulfides [35–37]. Bader charge analysis indicates that approximately 0.31 electrons were transferred to the W atom (Fig. S13). Compared with the individual components of WB and WC (Fig. S14), the shift of its W spectrum was at an intermediate level. WC showed this most obviously, which was basically in line with the results of theoretical calculations. Figure S15a shows the comparison of the XPS spectra of B 1*s*. Two chemical states, B–O and B–W, could be identified. Among them, the B–O peak might originate from the surface oxidation [38, 39]. After the LiPSs adsorbing, the B peak shifted to the lower energy region overall. This shift in binding energy was attributed to the fact that during the adsorption process, the B atom participated in charge transfer as an electron-deficient entity. In the XAFS Fourier transform spectra (Fig. 2i), a new W–S characteristic peak appeared in the low R region (~ 2.0 Å) of WB@WC-LiPSs, corresponding to the first shell of WS_2_. This indicated that a W–S coordination structure was formed on the WB@WC surface, further verifying that the W site in the heterostructure was involved in the bonding with sulfur atoms [40]. This result was also consistent with the XPS result of S 2*p* (Fig. S15b) [41]. The local environment of the W atom was reconstructed due to the interaction of polysulfides, which would provide new active sites for catalytic reactions [42].

Specifically, the adsorption and catalytic activity of the catalyst are jointly determined by the W-*d* orbitals of the metal center and the S-*p* orbitals of LiPSs [43]. It can be seen from the results of the single component of WB that the overlap degree of S-*p* orbitals and W-*d* orbitals in the WB@WC heterogeneous interface was higher (Fig. S16a, b), indicating that the introduction of WC enhanced the interaction between LiPSs and the catalytic substrate. The *d*-orbitals of metal W-based catalysts can be classified into dxy, dxz, dzy, dz^2^_,_ and dx^2^-y^2^ orbital states, and can form bonds with the S-*p* orbitals, thereby regulating the catalytic activity of the catalyst. Figure S16c**–**e calculates and presents the energy level arrangement of the five *d*-orbitals of W and the three *p*-orbitals of S. The projected density of states (PDOS) shows that the dz^2^, dxz, and dx^2^-y^2^ orbitals of W exhibited the maximum energy level overlap with the px, py, and pz orbitals of S, respectively.

Figure S17 shows the SEM cross-sectional morphology of the WB@WC-modified PP separator. The catalyst was uniformly coated on the separator surface, and the measured thickness was only ~ 6.7 μm, which was lower than that of most separator coating layers [44–47]. To explore the inhibitory effect of WB@WC coating on dissolved LiPSs, a visual diffusion experiment was conducted using an H-type electrolytic cell (Fig. S18). After standing for 12 h, it was observed that the right chamber solution turned significantly yellow in the unmodified PP system, while only slight discoloration occurred in the WB@WC-modified separator. This directly confirms that the WB@WC coating can effectively block the shuttling of LiPSs. In addition, the contact angle test (Fig. S19) shows that the contact angle of the WB@WC-based separator was the lowest, indicating that it can provide the best affinity with the electrolyte and thus favorable conditions for the mass transfer process [48].

### Catalytic Kinetics Analysis

**Fig. 3:**
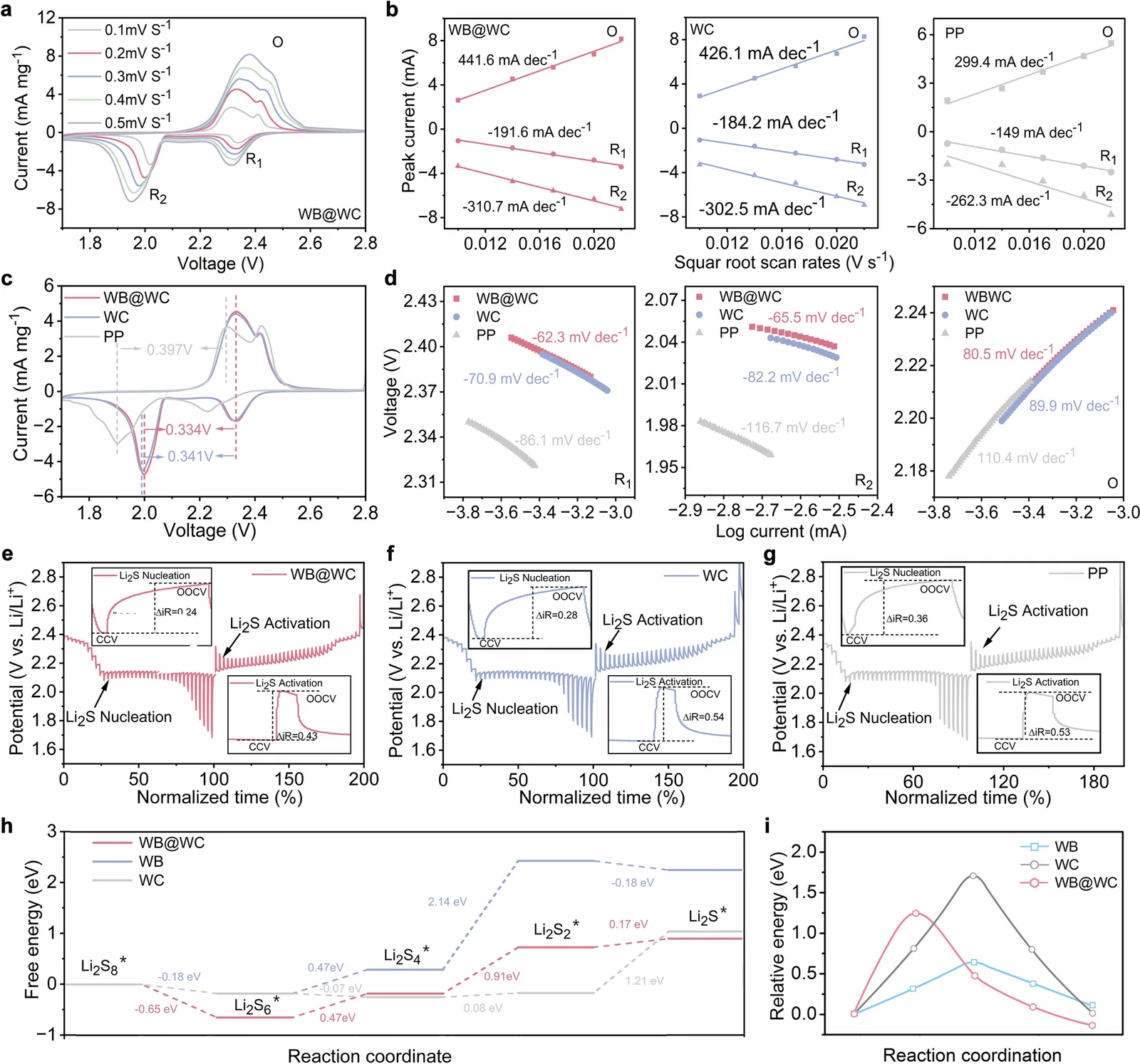
Dynamic testing and theoretical calculation. **a** CV of as-obtained WB@WC-based cathode at various scan rates. **b** Linear fitting comparison of peak currents in different CV curves. **c**,** d** CV curves and corresponding Tafel fitted slopes of the WB@WC, WC, and pure PP-based cathodes at 0.1 mV s^−1^. **e–g** GITT voltage curves of three different cathodes at 0.1 C. **h** Gibbs free energy for the sulfur species reduction process on WB or WC or WB@WC. **i** Decomposition energy barriers of Li_2_S on different substrates obtained through theoretical calculation

The CV curves at scan rates ranging from 0.1 to 0.5 mV s^−1^ were tested (Figs. 3a and S20a–c). The results showed that all cathodes exhibited typical Li–S redox peaks, that is, two distinguishable cathodic peaks (R1, R2) and one wider anodic peak (O). Figures 3b and S20d show the linear fitting results of the square root of the scanning rate and the peak current. According to the Randles–Sevcik equation, the slope of the fitting curve is positively correlated with the Li-ion diffusion coefficient [27]. A higher slope indicates a faster Li-ion diffusion rate, which is more conducive to the kinetic conversion of LiPSs. At the oxidation peak and reduction peaks, the fitting slopes of WB@WC were 441.6, − 191.6, and − 310.7 mV dec^−1^, respectively, all of which were higher than those of WB, WC, and PP. This was consistent with the results in the low-frequency region of the EIS, further confirming the promoting effect of WB@WC on the Li-ion diffusion kinetics (Fig. S21). In the GITT tests in Figs. 3e–g, S22, and S23, the nucleation and activation internal resistances of Li_2_S during WB@WC discharge–charging were 0.24 and 0.43 Ω, respectively, which were lower than those of the cathode with other separators. In particular, it inherited the low Li_2_S activation resistance of pure WB, and this result was also consistent with theoretical calculation (Fig. 3i). It indicates that WB@WC effectively accelerated the migration of Li-ions in the electrode solid, thereby promoting the oxidation and reduction processes of Li_2_S/Li_2_S_2_ [49, 50].

The CV curves of sulfur cathodes assembled with different separators are compared in Figs. 3c and S24. The WB@WC-based cathode showed the highest peak current response, which indicates that it obtained a more significant electrochemical transfer process. Furthermore, its polarization voltage was only 0.334 V, significantly lower than that of WC (0.341 V), WB (0.370 V), and PP (0.397 V), which also suggests its outstanding performance in dynamic [51]. Although its value was slightly lower than that of WB at the R1 and R2 peak positions, the overall minimum voltage polarization still demonstrated the advantage of the heterointerface. Even if the scanning rate increased, the WB@WC-based cathode still maintained low voltage polarization. Most significantly, the WB@WC exhibited that the Tafel slopes at both the anodic and cathodic reactions were lower than those at WC and PP, which were approximately 62.3 mV dec^−1^ (R1), 65.5 mV dec^−1^ (R2), and 80.5 mV dec^−1^ (O), respectively (Fig. 3d). These values indicate superior bidirectional catalytic activity of heterostructure. This rapid charge transfer was also verified by the smaller charge transfer resistance in the high-frequency region of EIS (Fig. S21), and by the more obvious current response and the lower redox overpotential in the CV of symmetrical cells (Fig. S25). Specifically, the charge transfer resistances of the four of them are ~ 9 Ω (WB@WC), ~ 12 Ω (WB), ~ 61 Ω (WC), and ~ 131 Ω (PP), respectively. DFT calculations further explored the catalytic capacity of the WB@WC heterogeneous interface from a theoretical perspective. As shown in Fig. 3h, during the overall liquid–solid conversion from Li_2_S_4_ to Li_2_S_2_/Li_2_S (rate-limiting step), WB@WC exhibited a lower free energy barrier, which was more conducive to nucleation [52]. The enhanced catalytic activity might result from defect site enrichment and electron delocalization at the WB@WC heterointerface [22, 53]. Overall, the above results all indicate that the 2D WB@WC nanosheets accelerated the Li–S reactions in terms of rapid adsorption of LiPSs, reduction in charge transfer energy barriers, and optimization of Li-ion kinetic pathways.

### Electrochemical Performance of Li–S Batteries

**Fig. 4:**
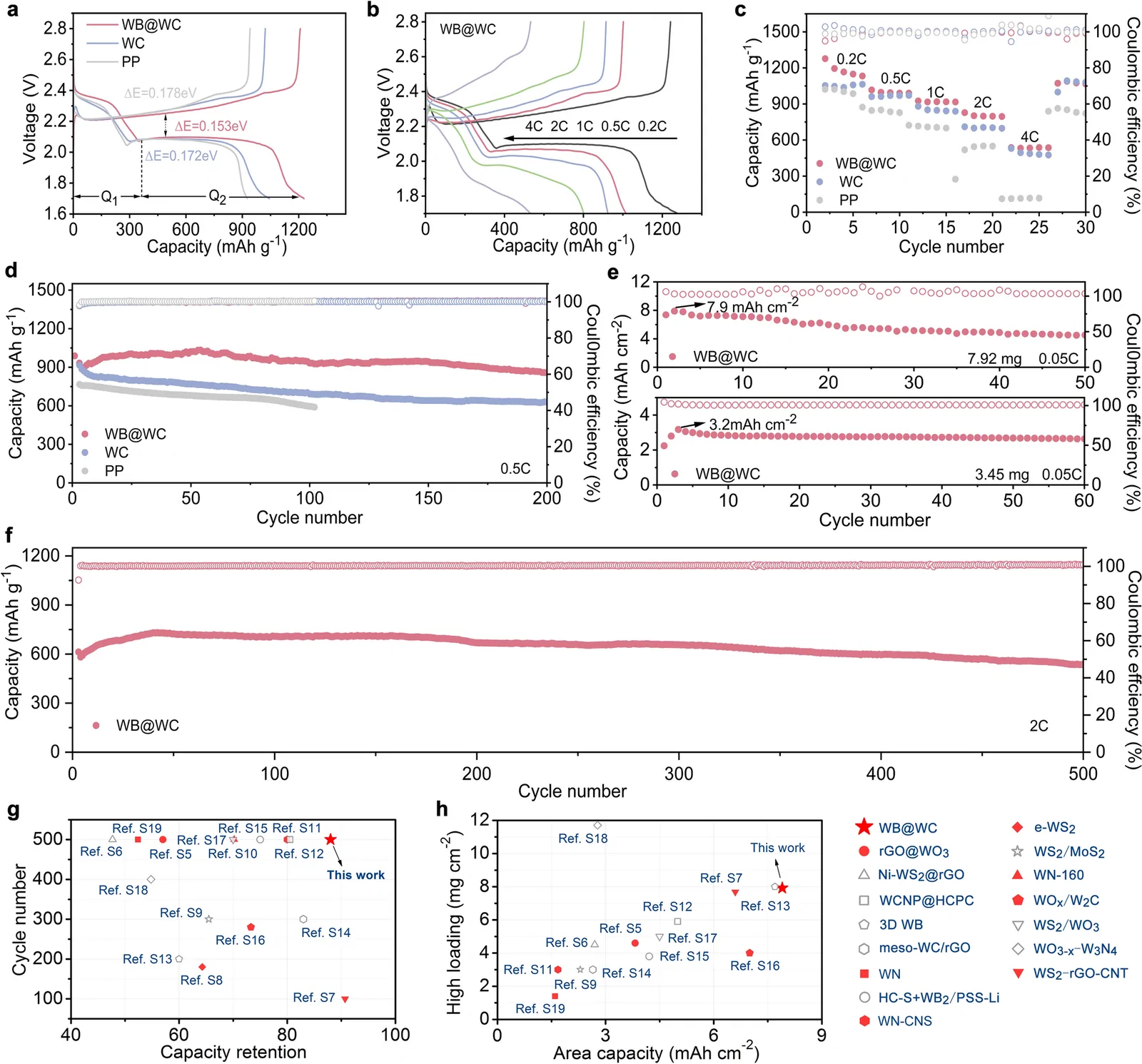
Electrochemical performance. **a** Comparison of galvanostatic charge–discharge profiles based on different separators at 0.2 C. **b** Charge–discharge profiles of cathode with WB@WC-modified separator at various rates. **c** Rate and **d** cycling performances of WB@WC, WC, and pp separators. **e** Cyclic data under high sulfur loading conditions. **f** Higher rate and longer cycle stability of WB@WC-based cathode. Comparison of the improvement of electrochemical performance by W-based materials in Li–S batteries. **g** Cycle number and capacity retention. **h** High loading and area capacity

To further explore the unique electrochemical properties of various modified separators, button Li–S batteries with Li metal as the anode and CNT/S as the cathode were assembled. The voltage profiles presented by the modified separators under various rates all showed standard Li–S charge–discharge platforms (Figs. 4b and S26a, b). In contrast, the battery with ordinary PP separator exhibited a plateau-free curve under high current density, indicating poor reaction kinetics (Fig. S26c). The corresponding curves at a low rate of 0.2 C are shown in Figs. 4a and S27, where Q_1_ corresponded to the solid–liquid conversion process from S_8_ to Li_2_S_4_, and Q_2_ corresponded to the liquid–solid conversion process from Li_2_S_4_ to Li_2_S/Li_2_S_2_ [34]. Obviously, compared with WC and PP, the WB@WC-modified separator showed the smallest voltage difference. Furthermore, as shown in Fig. S28, the Q_2_ to Q_1_ ratios of WB@WC, WC, WB, and PP-based batteries were 2.51, 2.42, 2.40, and 2.16, respectively, indicating that the WB@WC most effectively promoted the conversion of more active sulfur species to the final products, which was conducive to the increase of discharge depth. Figure S26e, f compares the nucleation overpotential and decomposition overpotential of Li_2_S/Li_2_S_2_. The battery based on WB@WC not only exhibited a significantly reduced nucleation overpotential (~ 13 mV) but also achieved a higher capacity (~ 349 mAh g^−1^) at the first discharge plateau. In addition, the WB@WC-optimized cell showed the lowest Li_2_S/Li_2_S_2_ decomposition overpotential (2.23 V). WB also exhibited a very low decomposition overpotential (2.24 V), which was relatively consistent with the activation resistance of Li_2_S determined by GITT. This might be due to the low reaction energy barrier of this process. This is also consistent with the results of the Li_2_S decomposition energy barrier obtained through theoretical calculation (Fig. 3i). A more efficient Li_2_S/Li_2_S_2_ conversion–decomposition process would effectively reduce the content of “dead sulfur,” thereby improving the overall performance [54]. The smooth realization of liquid–solid and solid–liquid conversions indicates that the MBene heterostructure exhibited the bifunctional catalysts function.

As the rate increased from 0.2 to 4 C, the initial capacity of the cell equipped with WB@WC-modified separator reached 1277, 1016, 925, 828, and 538 mAh g^−1^_**,**_ respectively (Fig. 4c). Even after repeated rate switching back to 0.2 C, it was still able to achieve a reversible capacity of 1072 mAh g^−1^. In contrast, the rate performance of WC and WB modified separators was significantly inferior (Fig. S26d). Notably, the battery using an unmodified separator almost completely lost its discharge capacity under high current density. These results fully confirm that the “adsorption–migration–catalysis” synergistic mechanism of LiPSs was optimized through interfacial electronic regulation, and the WB@WC provided a positive impact on the reaction kinetics and stability of the sulfur cathode.

Subsequently, further cyclic stability was explored. As shown in Fig. 4d, after 200 cycles at 0.5 C, WB@WC still maintained 858 mAh g^−1^, with a capacity retention rate as high as ~ 92% (Coulombic efficiency approached 100%), which was much higher than the ~ 68.8% of WC and ~ 69.7% of WB (Fig. S29). However, the capacity of the cell using the original PP dropped rapidly to 594 mAh g^−1^ after only 100 cycles. When the current density increased to 2 C, the WB@WC electrode, after a brief activation, could maintain stable capacity up to 500 charge–discharge cycles (Fig. 4f). Starting from the first cycle, the capacity fading rate per cycle was only about 0.024%, showing excellent cycling stability. Figure S30 shows the SEM characterization images of the surface of the Li anode after cycling. The results showed that the surface of the Li anode of the WB@WC-modified battery was flatter and smoother, while the surface of the Li anode of the battery with pure PP separator was full of pores and the roughness was significantly increased. The occurrence of this difference was speculated to be closely related to the side reactions of shuttle LiPSs and the inhomogeneity of the Li deposition [55, 56].To meet the demands of commercial applications, batteries also need to possess outstanding electrochemical performance under conditions of high sulfur loading. As shown in the Fig. 4e, when the sulfur loading was increased to 3.45 mg cm^−2^, the initial area capacity reached 3.2 mAh cm^−2^ and maintained outstanding stability in the subsequent cycle. After further increasing the sulfur loading to 7.92 mg cm^−2^, the initial capacity was as high as 7.9 mAh cm^−2^, and it remained at 4.53 mAh cm^−2^ after 50 cycles. Figure S31 presents the charge–discharge curves under different high loadings, which clearly shows that it exhibited the characteristics of Li–S platforms, meaning that even at such a high sulfur content, the battery with WB@WC coating still catalyzed the progress of multi-electron reactions. These results fully demonstrated that WB@WC was indeed beneficial to the stability improvement. This was not only due to its effective mitigation of the shuttle effect, but also attributed to its excellent catalytic activity for the LiPS conversion. Figure 4g, h compares the cycling stability and high-loading of the MBene-based cell in this work with other modified Li–S batteries. Obviously, 2D WB@WC demonstrated superior comprehensive performance compared to other previous W-based nanomaterials (Table S2).

### In Situ Characterizations

**Fig. 5:**
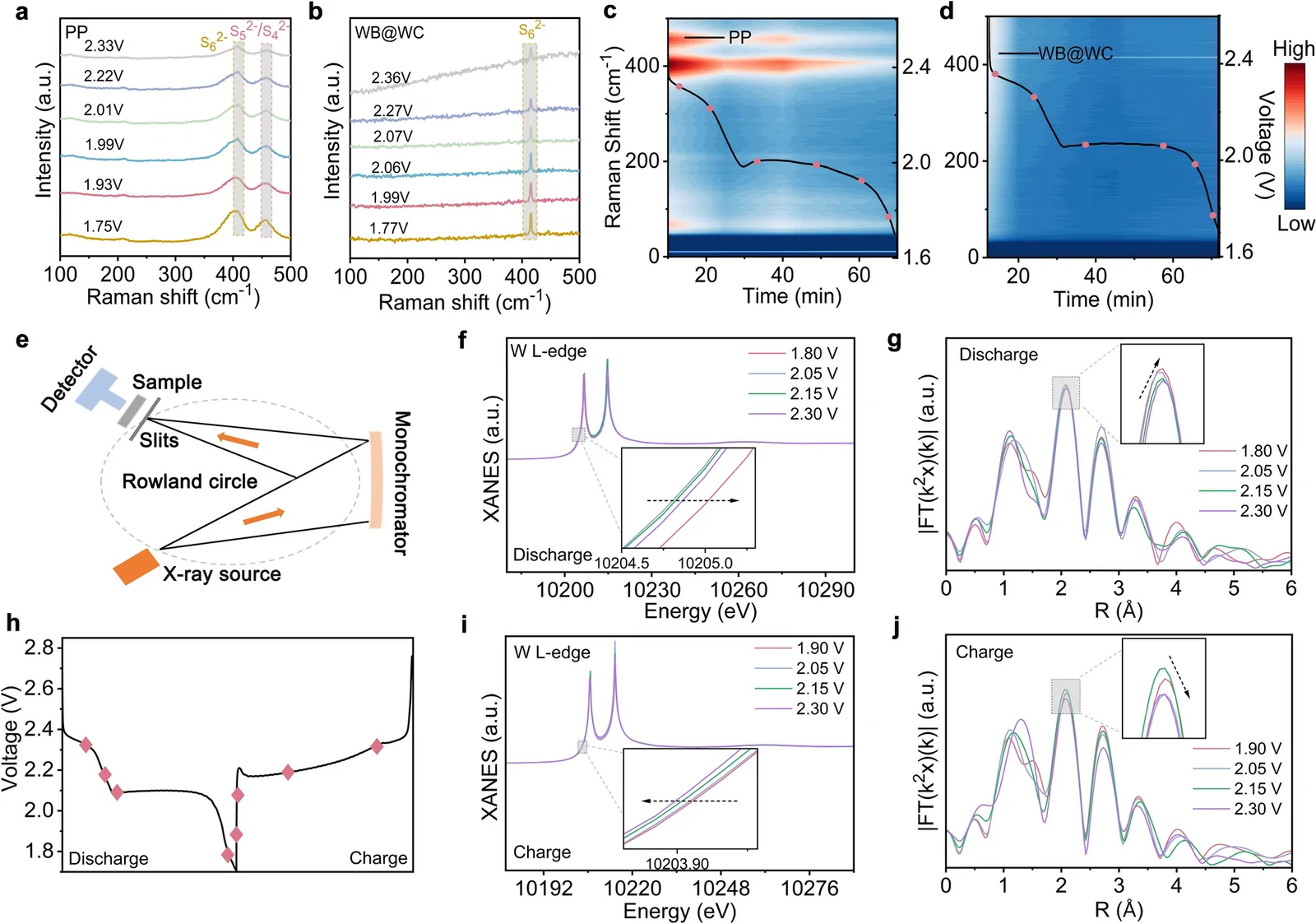
In situ characterization tests. **a**, **b** In situ Raman of Li–S cells based on pure PP separator and WB@WC-modified separator during the discharge process. **c**, **d** Corresponding planar time-resolved Raman spectra. **e** In situ XANES device. **f**, **i** In situ XANES curves and **g**, **j **In situ XAFS spectra of the WB@WC-based cell at different voltages. **h** In situ discharge–charge process

To further explore the effect of WB@WC-modified separator in inhibiting the shuttling of LiPSs, in situ Raman was carried out in this study. The corresponding in situ schematic diagram is shown in Fig. S32. As shown in Fig. 5a, c, for the cell with an unmodified separator, wide and significant characteristic peaks of S_6_^2−^ and S_5_^2−^/S_4_^2−^ were detected at ~ 406 and ~ 454 cm^−1^ as the reaction progressed, indicating that the soluble polysulfide intermediate was actively shuttled between the anode and cathode through the PP separator [57, 58]. In contrast, for the cell with the separator modified by the WB@WC catalyst (Fig. 5b, d), only a weak Raman characteristic peak corresponding to S_6_^2−^ was detected. This indicates that the WB@WC coating could strongly block the diffusion of LiPSs. The 3D diagram in Fig. S33 can present the comparison effect more intuitively. The occurrence of this phenomenon was attributed to the optimization of the LiPSs adsorption–catalysis synergistic mechanism by interface electron regulation, which not only accelerated the LiPS migration but also further promoted their reduction to Li_2_S.

In situ absorption spectrum monitoring further explored the electronic structure of the W element and the dynamic evolution of its local coordination environment during the LiPS reactions (Fig. 5e, h). Although experiments around the absorption edge position are uncertain, careful observation of its changes can still reveal some clues. As the discharge voltage gradually decreased to 1.80 V, the absorption edge of the XANES shifted toward the high-energy region overall, meaning an increase in the valence state of the W element (Fig. 5f) [59]. This phenomenon indicates that during the discharge process, W atoms underwent electron-losing behavior and actively participated in the reduction reaction of active sulfur species. During the charging process (Fig. 5i), the absorption edge gradually shifted back to the low-energy region, suggesting that the valence state of W dropped again. This proves that the W element gained electrons during this process, thereby driving the oxidative decomposition of Li_2_S_2_/Li_2_S [60]. Although W atoms accelerated the conversion of LiPSs through electron transfer, during the entire cycle, the changes in the slope of the absorption edge and the intensity of the white line peak in the XANES spectrum exhibited continuous and dynamic characteristics, with no obvious new peaks appearing. This indicates that the W sites in WB@WC did not undergo irreversible structural distortion, thereby ensuring the stability of their catalytic activity. From the in situ XAFS analysis (Figs. 5g, j and S34), it could be known that during the discharge–charge process, the peak intensity of the W–S shell was dynamically adjusted with the variation in voltage. During discharging, the strength of the W–S bond increased, which might be related to the dissociation of S atoms from LiPSs. During charging, the intensity of the W–S peak showed a shortening trend, indicating that S atoms recombined to form long-chain LiPSs [61]. This phenomenon seems to reflect the dynamic behavior of adsorption–conversion of LiPSs on the WB@WC surface.

## Conclusions

Herein, the WB@WC heterostructure was successfully constructed by in situ constructing WC nanocrystals on the surface of 2D WB-based MBene. As a modified medium for Li–S batteries, it efficiently realized the anchoring–migration–catalytic conversion process of LiPSs. On the one hand, C atom in WC showed a moderate electronegativity, which could assist in regulating the delocalization of the d-orbital electrons of W, enhancing the interfacial electrostatic interaction and LiPSs adsorption. The orbital overlap at the heterointerface of WC and WB built a channel for electrons, enhancing electron delocalization and constructing an internal electric field, thereby reducing the LiPSs reaction energy barrier in multiple aspects. On the other hand, 2D ultrathin materials played a significant role in reducing charge migration paths and enhancing active sites. Electrochemical performance tests show that WB@WC could significantly optimize battery kinetics, enabling the reversible capacity at 0.2 and 4 C to reach as high as 1277 and 538 mAh g^−1^, respectively. Even at a high sulfur loading (7.92 mg cm^−2^), the initial area capacity still reached 7.9 mAh cm^−2^. Meanwhile, In situ Raman directly confirmed the inhibitory effect of WB@WC on LiPSs shuttling. The attenuation rate within 500 cycles of capacity was only ~ 0.024% per cycle. More importantly, this study provides new insights for the structural regulation and functional optimization of MBene derivatives, while offering a universal reference for the design of boride heterocatalysts. This strategy can be extended to multi-component MBene heterogeneous systems in the future, exhibiting broad application potential in energy storage and catalysis.

## Supplementary Information

Below is the link to the electronic supplementary material.

Supplementary file1 (DOCX 10038 kb)
