# Supplementary material for: Facilitated Polysulfide Redox Conversion by Delocalized Electrons in MBene Heterointerface for Highly Stable Lithium–Sulfur Batteries
Source: Nanomicro Lett. 2026 Feb 11;18:252. doi: 10.1007/s40820-026-02100-3 (PMC12894609; doi:10.1007/s40820-026-02100-3)
Supplement: Supplementary file 1 — Supplementary file1 (DOCX 10038 kb) [file 40820_2026_2100_MOESM1_ESM.docx]

Supporting Information for

**Facilitated Polysulfide Redox Conversion by Delocalized Electrons in MBene Heterointerface for Highly Stable Lithium–Sulfur Batteries**

Guifen Wu^1^, Yunmiao Fan^3^, Jiatong Li^3^, Zhaoxi Shen^2,^* , Yuxiu Xie^1^, Peixun Yang^1^, Jun Pu^1,^*

^1^ Key Laboratory of Functional Molecular Solids (Ministry of Education), Anhui Provincial Engineering Laboratory for New-Energy Vehicle Battery Energy-Storage Materials, College of Chemistry and Materials Science, Anhui Normal University, Wuhu 241002, P. R. China

^2^ College of Chemistry and Materials Science, Key Laboratory of Analytical Science and Technology of Hebei Province, Institute of Life Science and Green Development, Hebei University, Baoding 071002, P. R. China

^3^ State Key Laboratory of Multiphase Complex Systems, Institute of Process Engineering, Chinese Academy of Sciences, Beijing 100190, P. R. China

*Corresponding authors. E-mail: [jpu@ahnu.edu.cn](mailto:jpu@ahnu.edu.cn) (Jun Pu); [zhaoxishen@hbu.edu.cn](mailto:zhaoxishen@hbu.edu.cn) (Zhaoxi Shen)

**S1 Experimental Section**

**Theoretical calculation:** The electronic properties and optimize geometries of all the investigated structures in this work were calculated based on density functional theory (DFT). The cambridge sequential total energy package (CASTEP) was employed with a general gradient approximation and Perdew-Burke-Ernzerh of exchange correlation functional [S1, S2]. A plane-wave cutoff energy of 500 eV was employed for the standard norm-conserving pseudopotentials [S3]. The Brillouin zone was sampled with Monkhorst-Pack mesh k-points of 3×3×1 grid. A vacuum slab thickness of 15 Å was set between slabs along the Z-axis. The binding energy (E) between the material and polysulfides was calculated as follows: E = (E_mat_ + E_ps_) – E_mat+ps_, where E_mat_, E_ps_, and E_sub+ps_, denoted the energy of the materials, polysulfides and total binding system, respectively [S4].

**S2 Supplementary Figures and Tables**


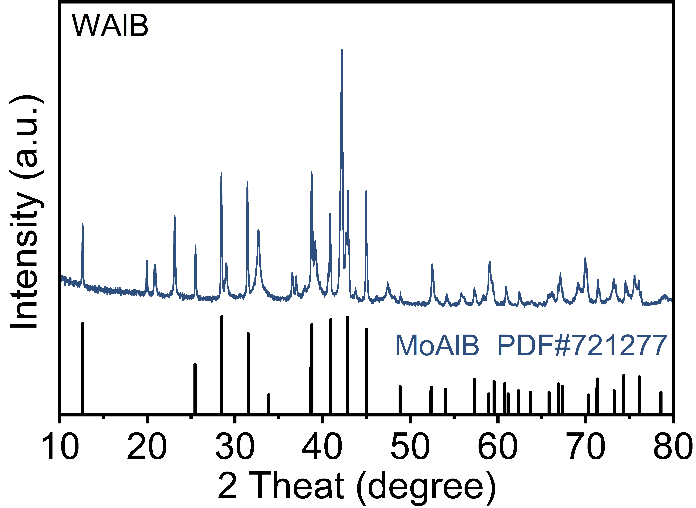


**Fig. S1** XRD of WAlB precursor


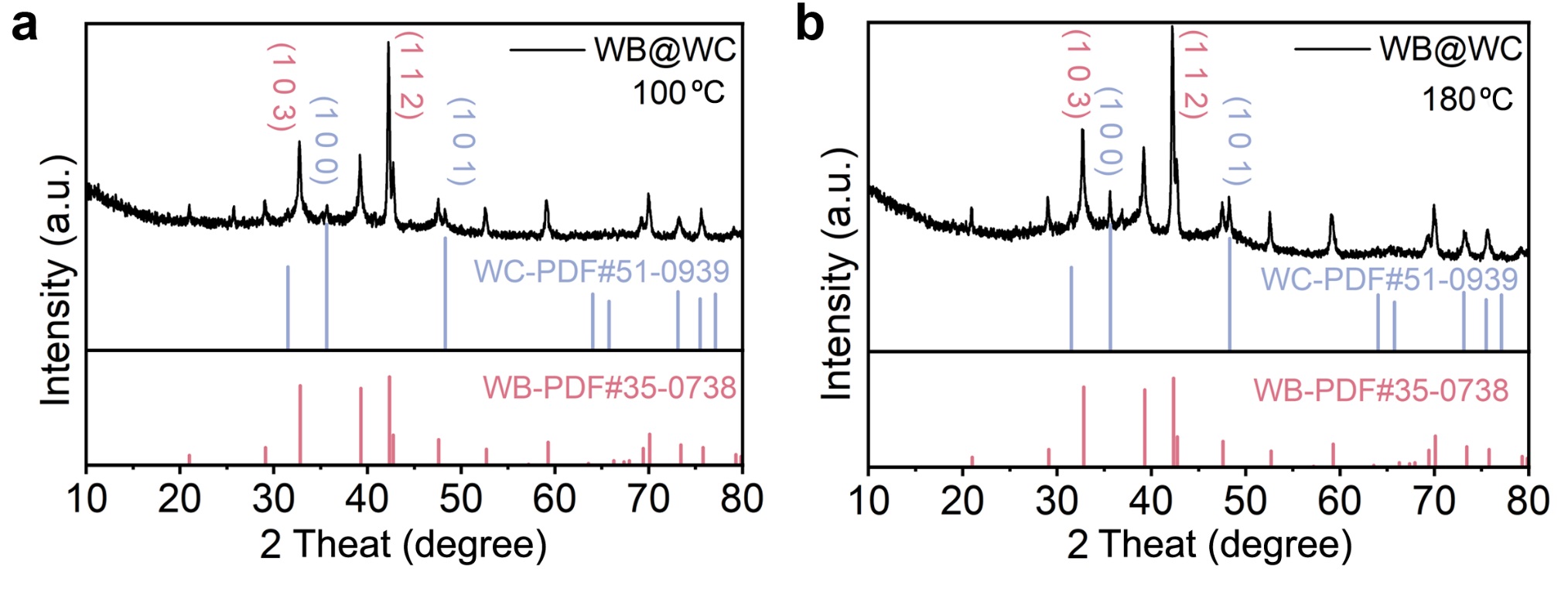


**Fig. S2** XRD results of WB@WC samples under different carbonization temperatures: **a** 100 °C; **b** 180 °C


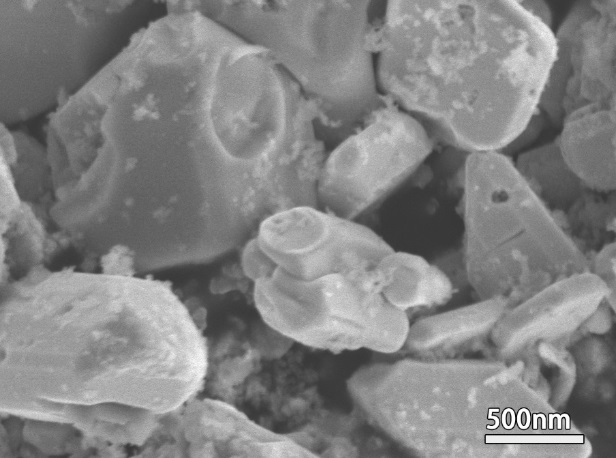


**Fig. S3** SEM of WAlB precursor with blocky structure


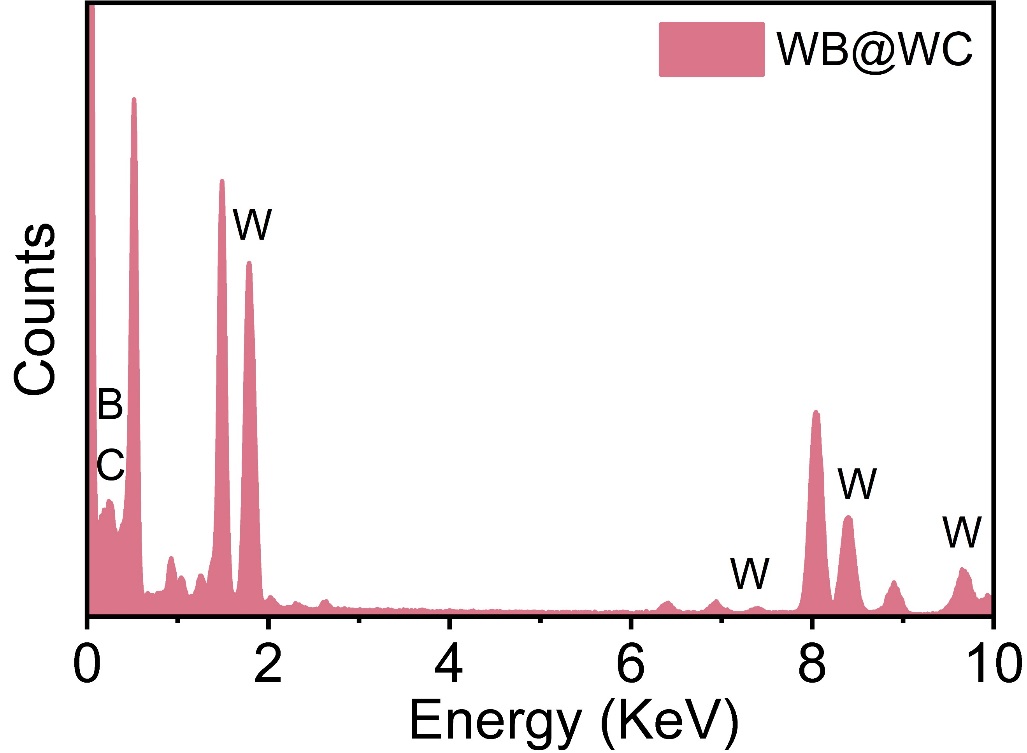


**Fig. S4** EDS of WB@WC heterostructures


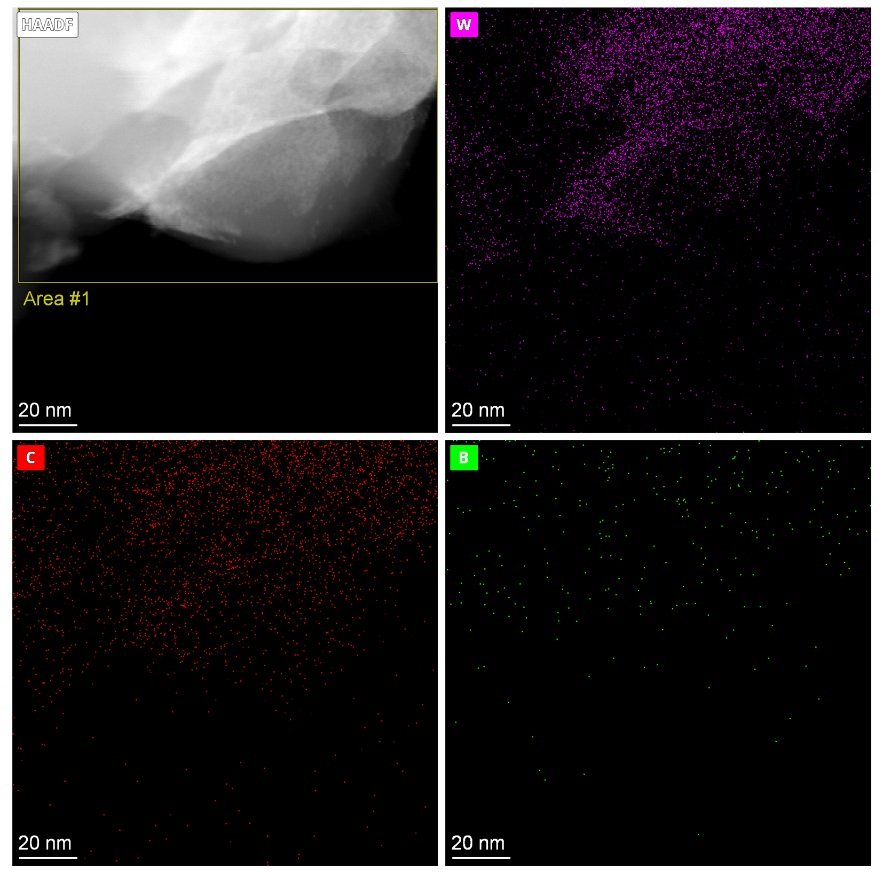


**Fig. S5** HAADF-STEM base elemental mapping with further magnification


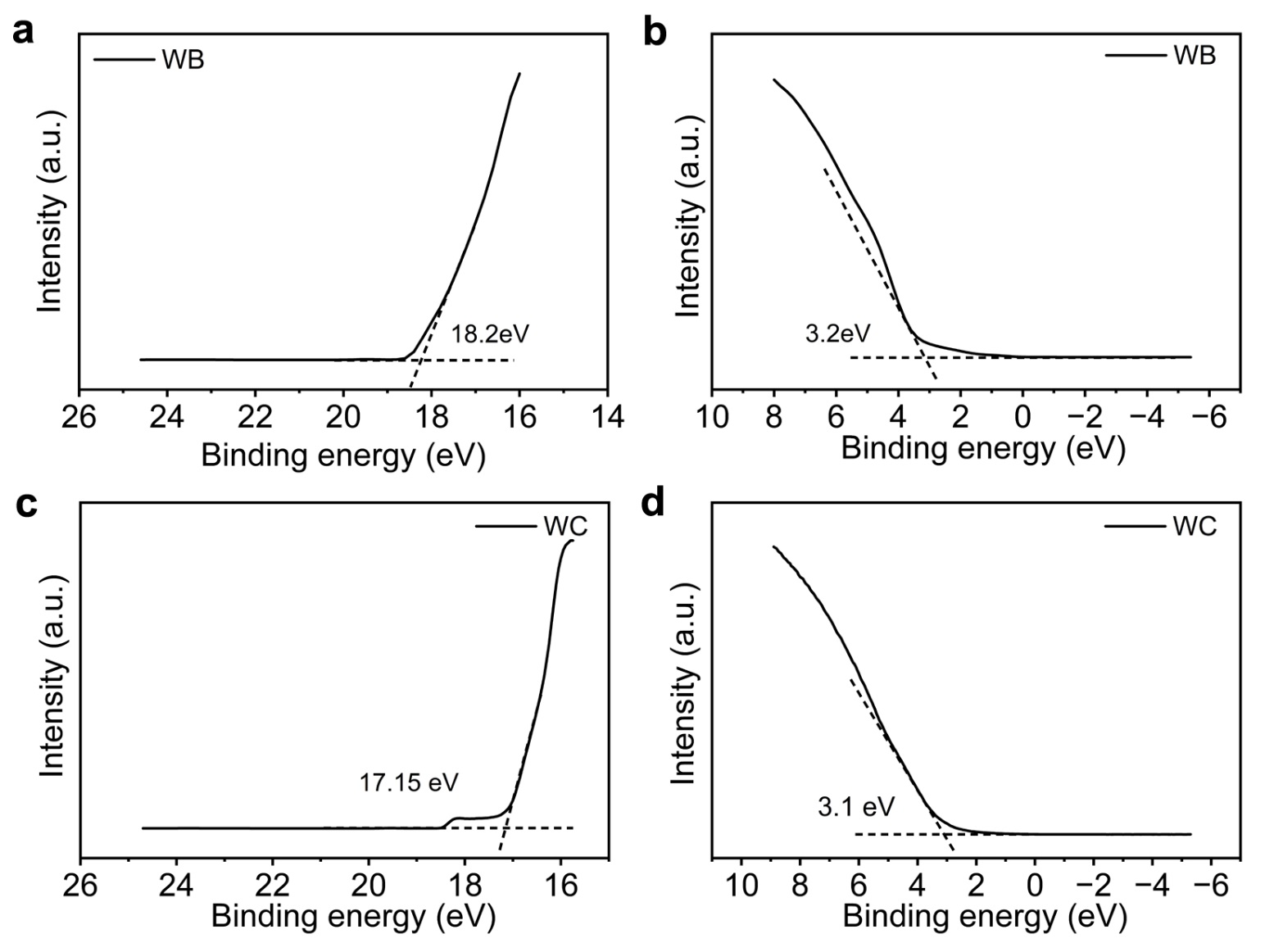


**Fig. S6** Ultraviolet photoelectron spectroscopy: **a** WB, and **b** WC

WB: Work function (Φ) = 21.2 – 18.2 = 3.0 eV VB/HOMO = 3.2 eV

WC: Work function (Φ) = 21.2 – 17.15 = 4.05 eV VB/HOMO = 3.1 eV


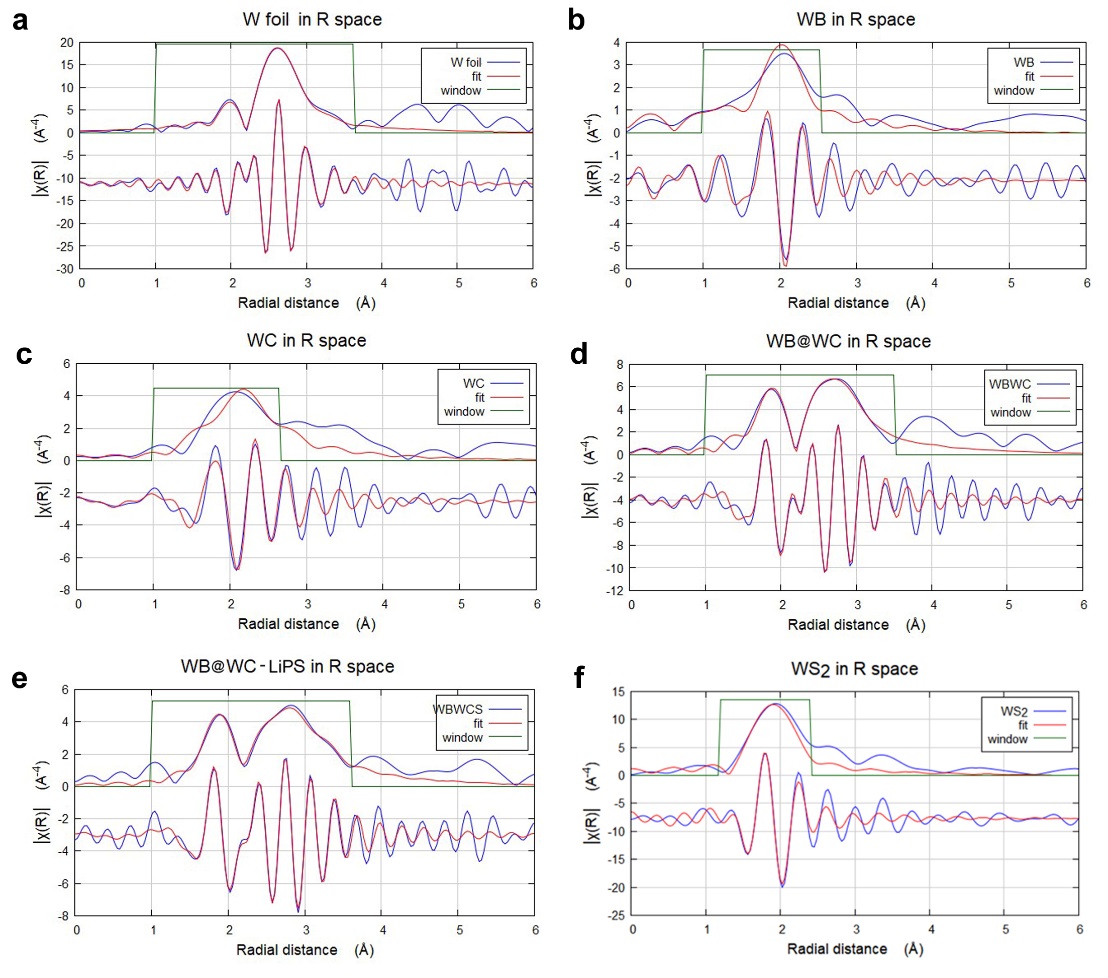


**Fig. S7** Fitting results of the XAFS spectra of different samples at k-space and R space: **a** W foil; **b** WB; **c** WC; **d** WB@WC; **e** WB@WC-LiPS; **f** WS_2_


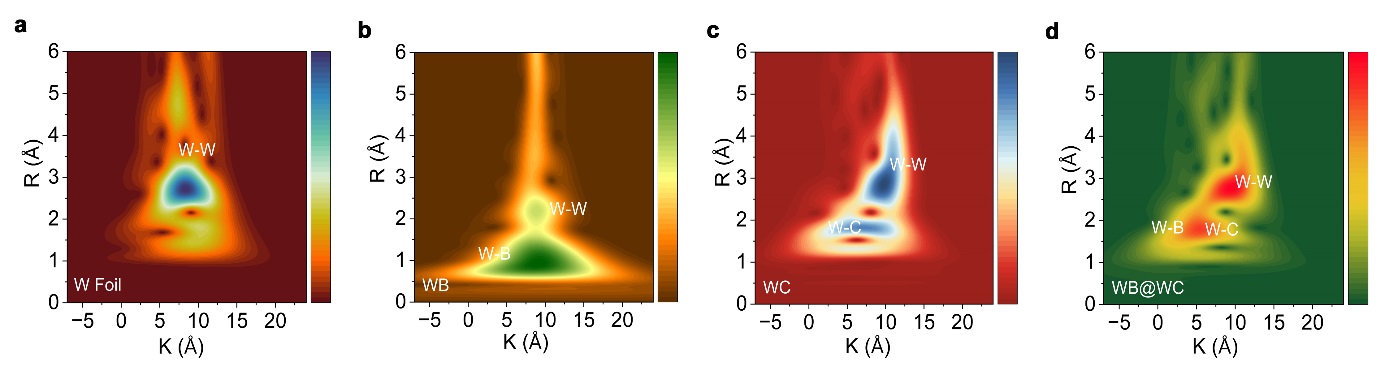


**Fig. S8** WT-XAFS of W L-edge for different samples


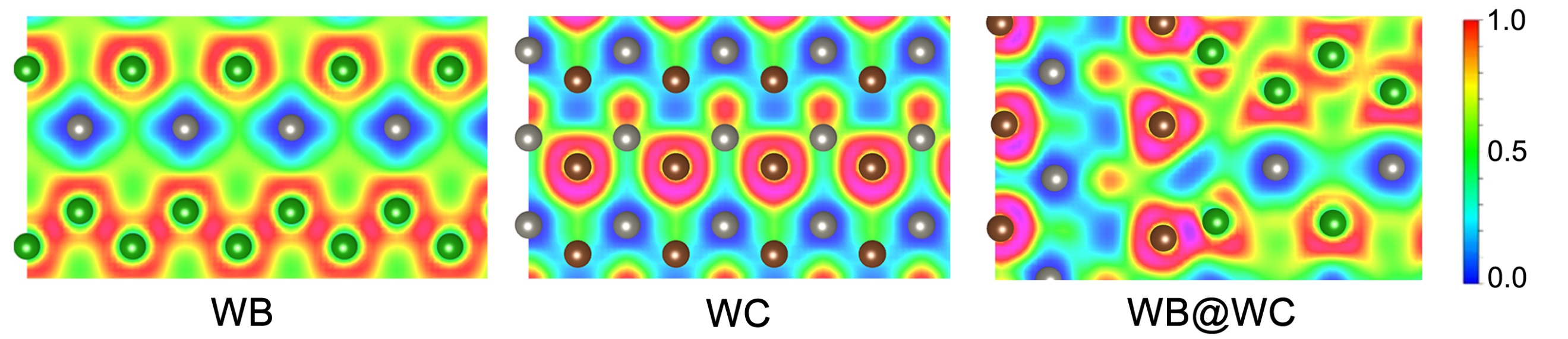


**Fig. S9** Calculation of electronic local function of WB, WC and WB@WC


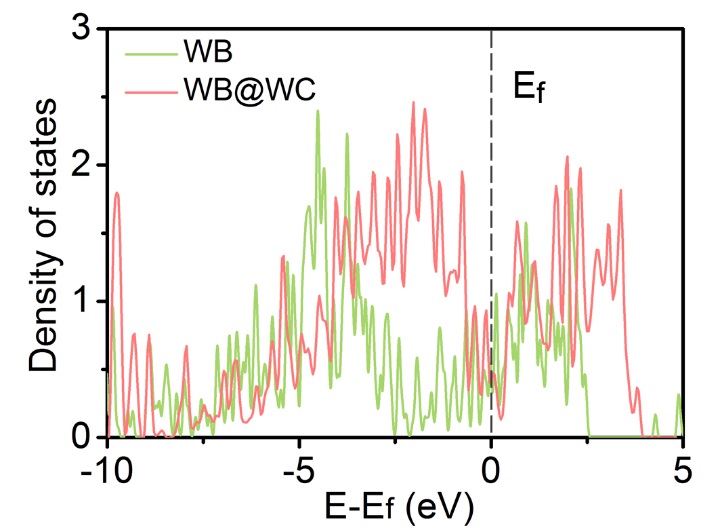


**Fig. S10** Density of states of WB and WB@WC


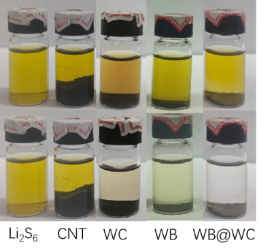


**Fig. S11** Associated color change of Li_2_S_6_ solution before and after adding different materials


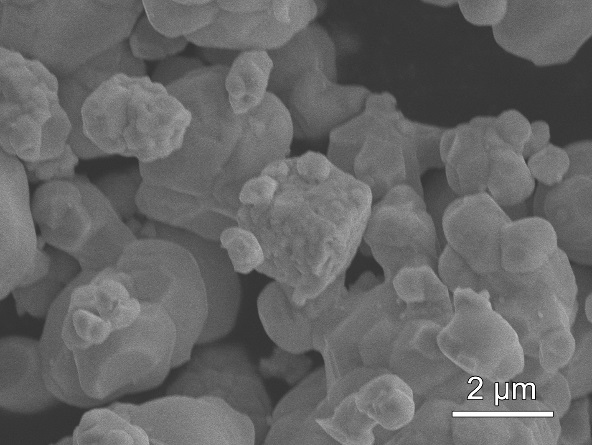


**Fig. S12** SEM of WC sample


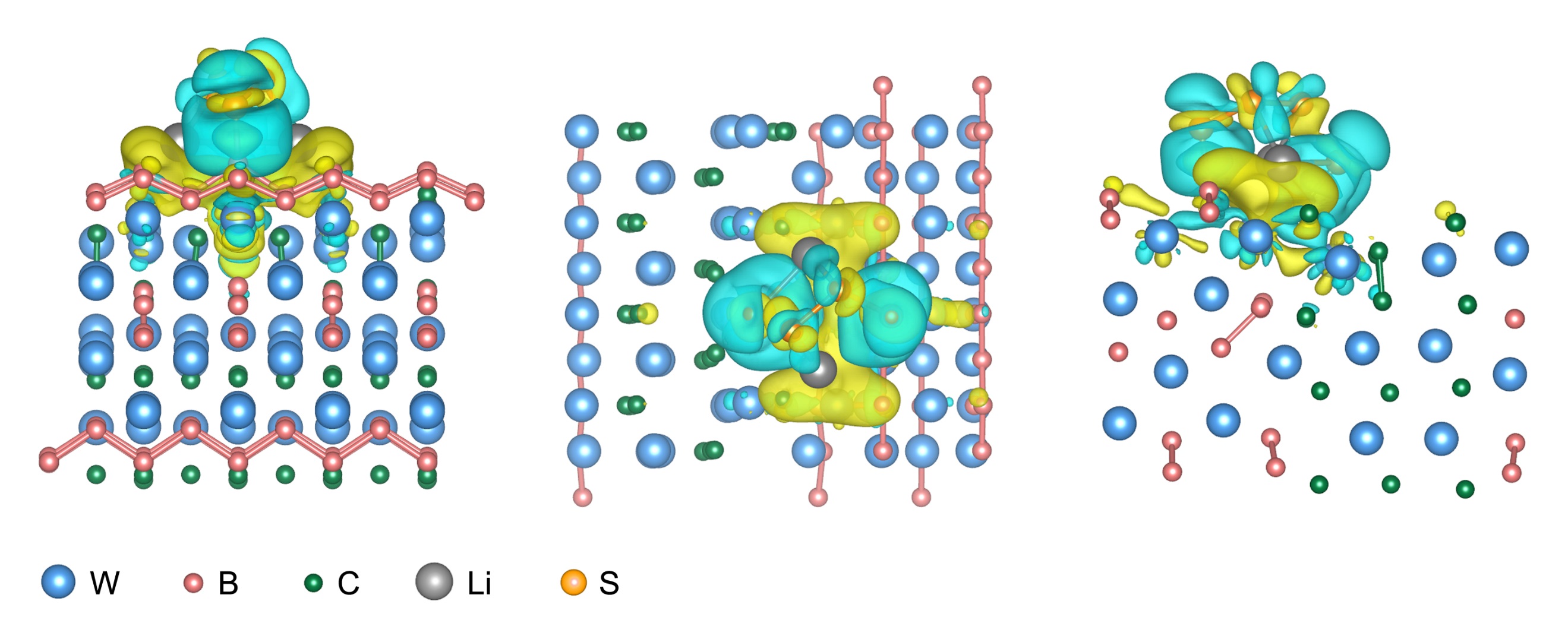


**Fig. S13** Bader charge analysis of WB@WC adsorbing LiPS at different angles


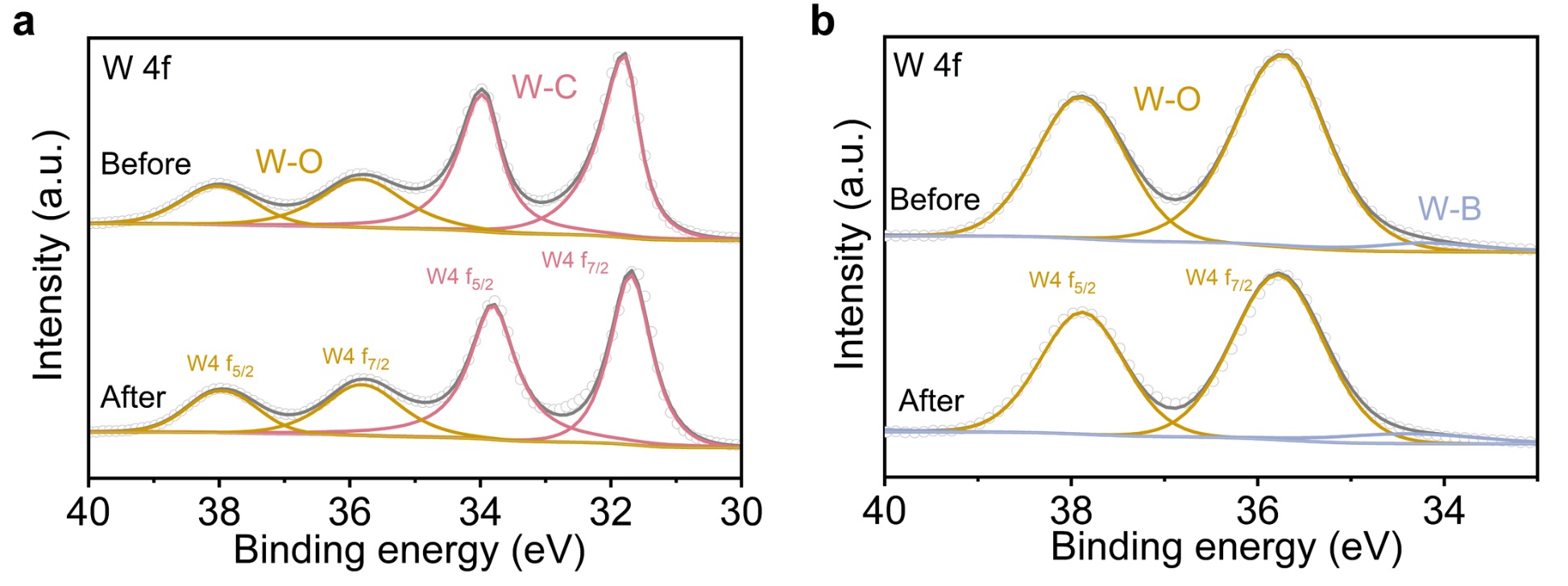


**Fig. S14** XPS of W4f before and after LiPSs adsorption


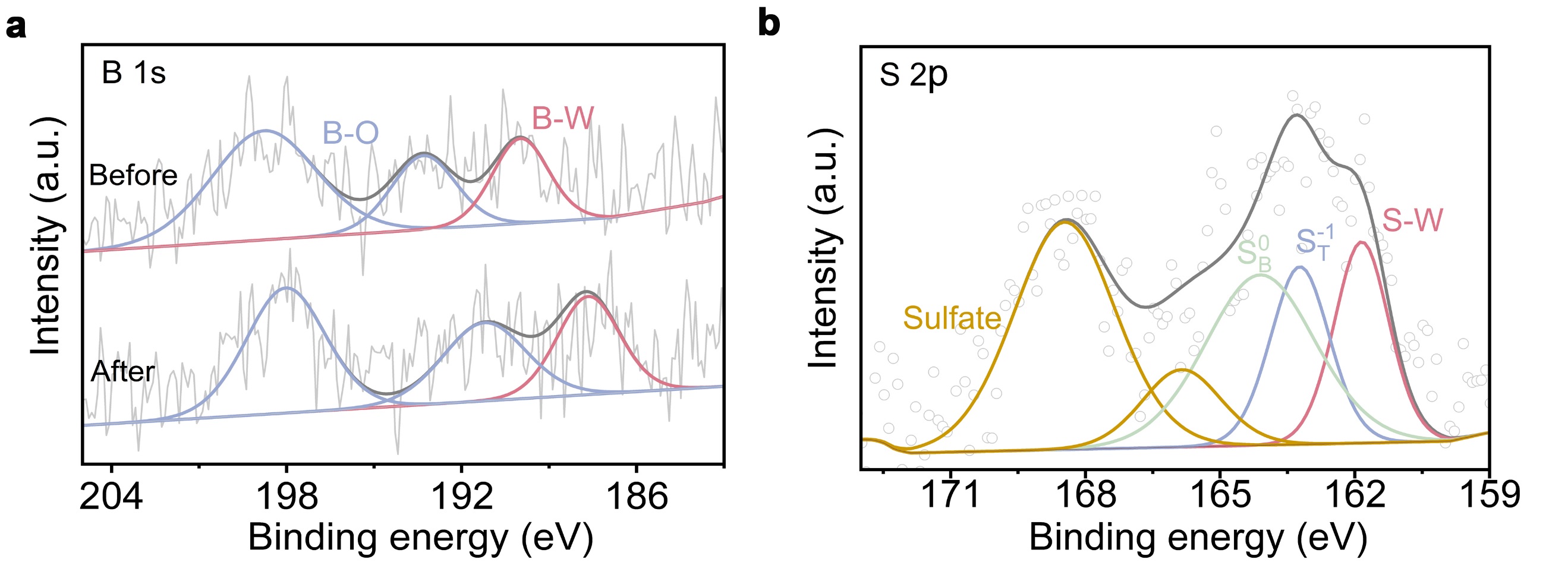


**Fig. S15** **a** XPS comparison of W element in WB@WC before and after adsorption of Li_2_S_6_. **b** XPS of S element in WB@WC after adsorption


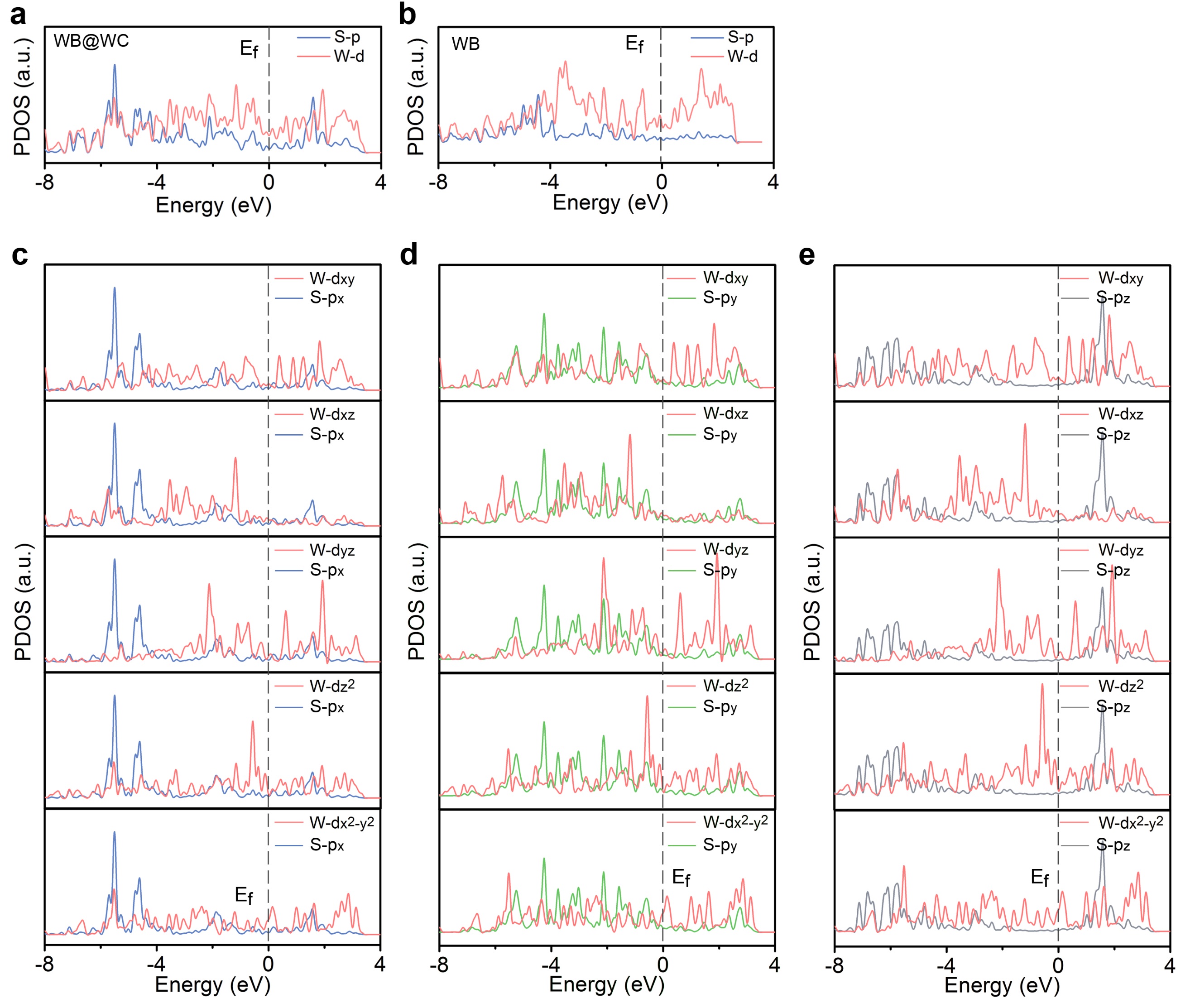


**Fig. S16** PDOS of for W-d orbital and S-p orbital the Li_2_S_6_ adsorbed on different materials: **a** WB@WC; **b** WB. Detailed information for the Li_2_S_6_ adsorbed on WB@WC: **c** W-d orbital and S-px orbital; **d** W-d orbital and S-py orbital; **e** W-d orbital and S-pz orbital


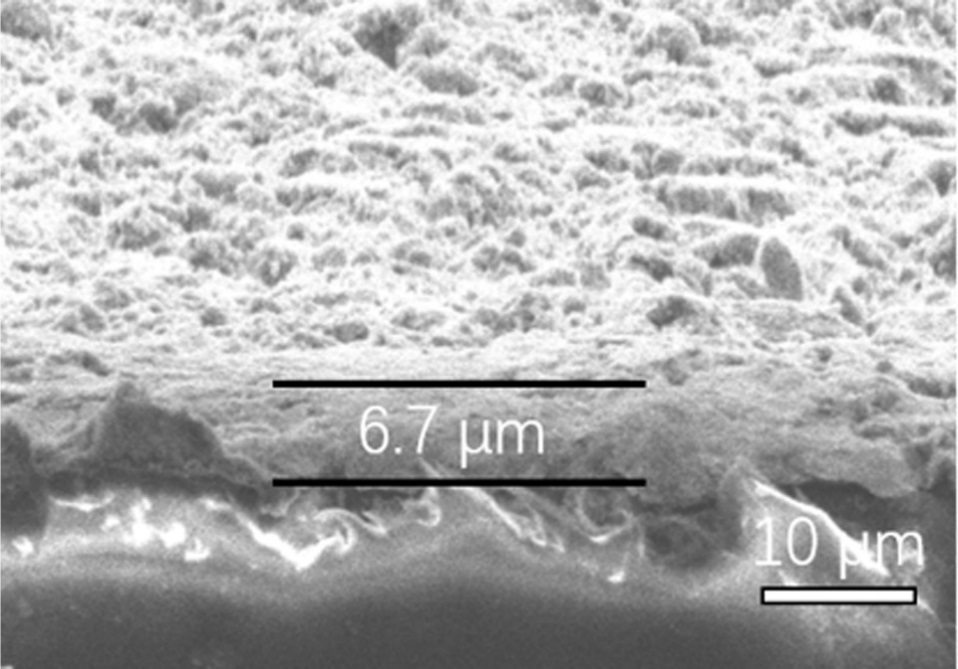


**Fig. S17** Cross-sectional SEM image of WB@WC coating layer on PP separator


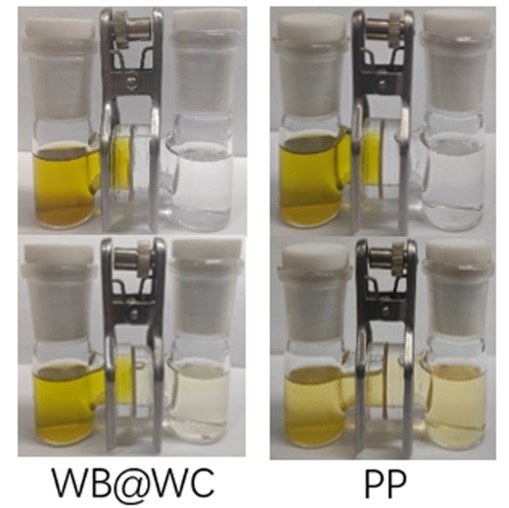


**Fig. S18** Visualized soluble Li_2_S_4_ penetration with H-type electrolytic cell


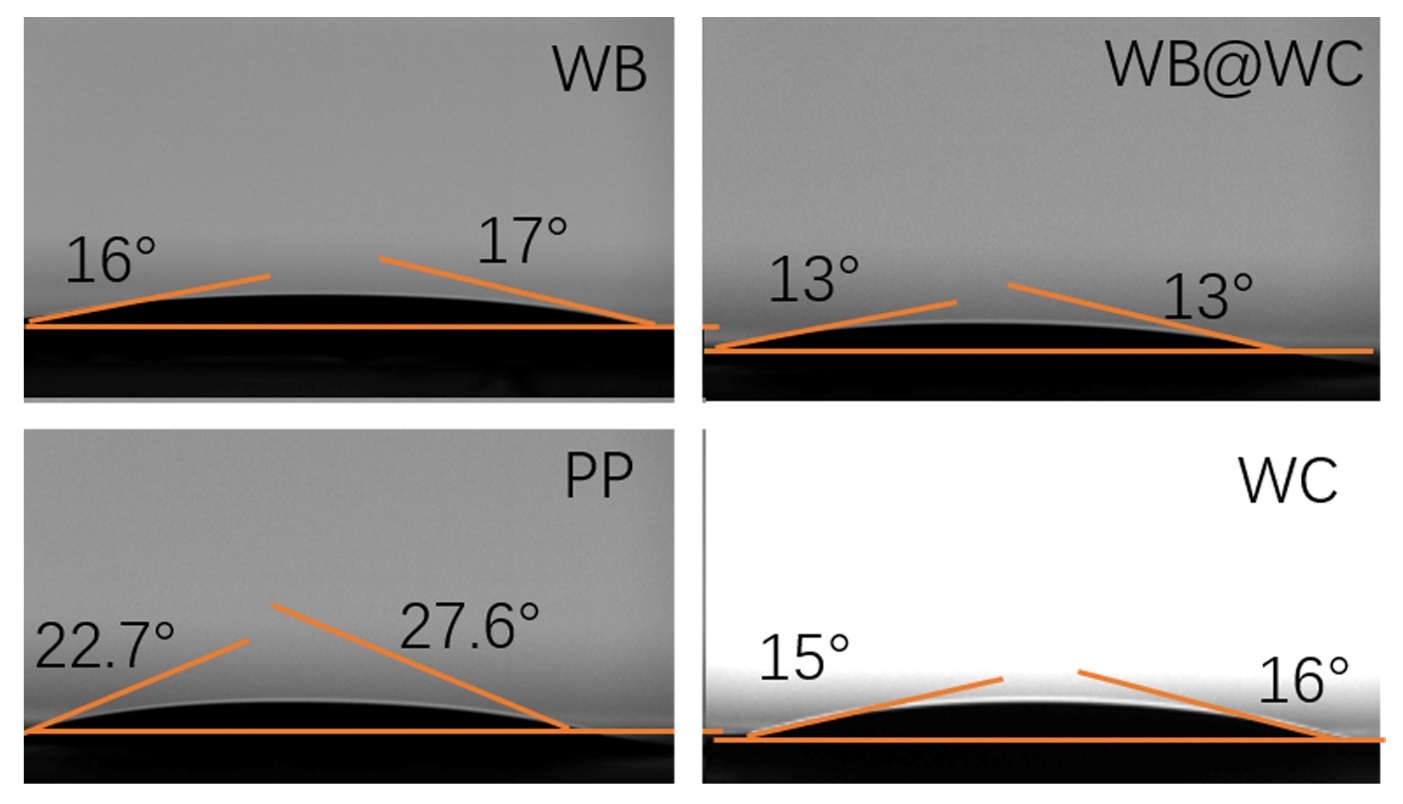


**Fig. S19** Optical images of contact angle measurements of Li**–**S electrolyte on different separators
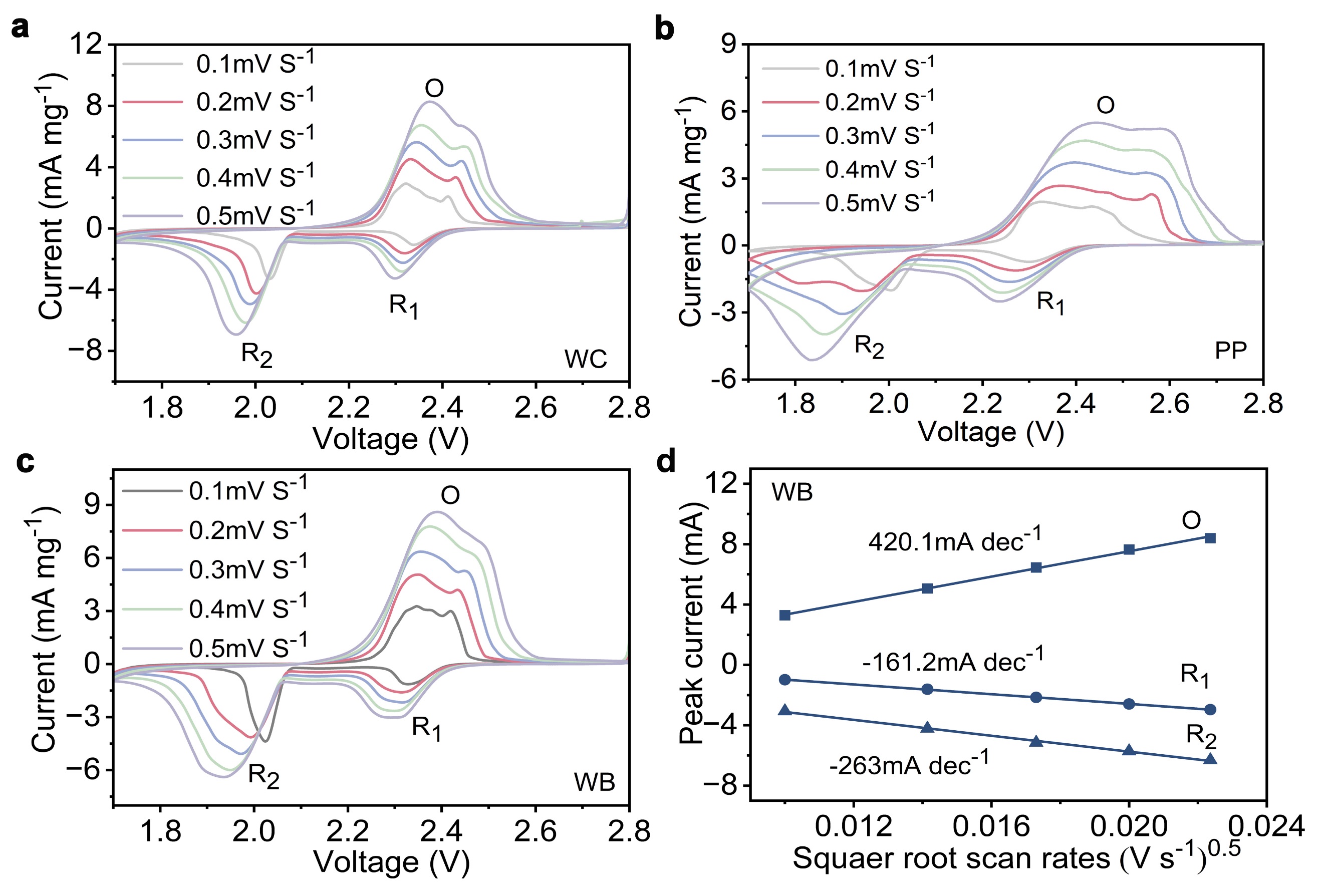


**Fig. S20** **a–c** CV of different separators at various scan rates. **d** Linear fitting comparison of peak currents in WB based CV curves


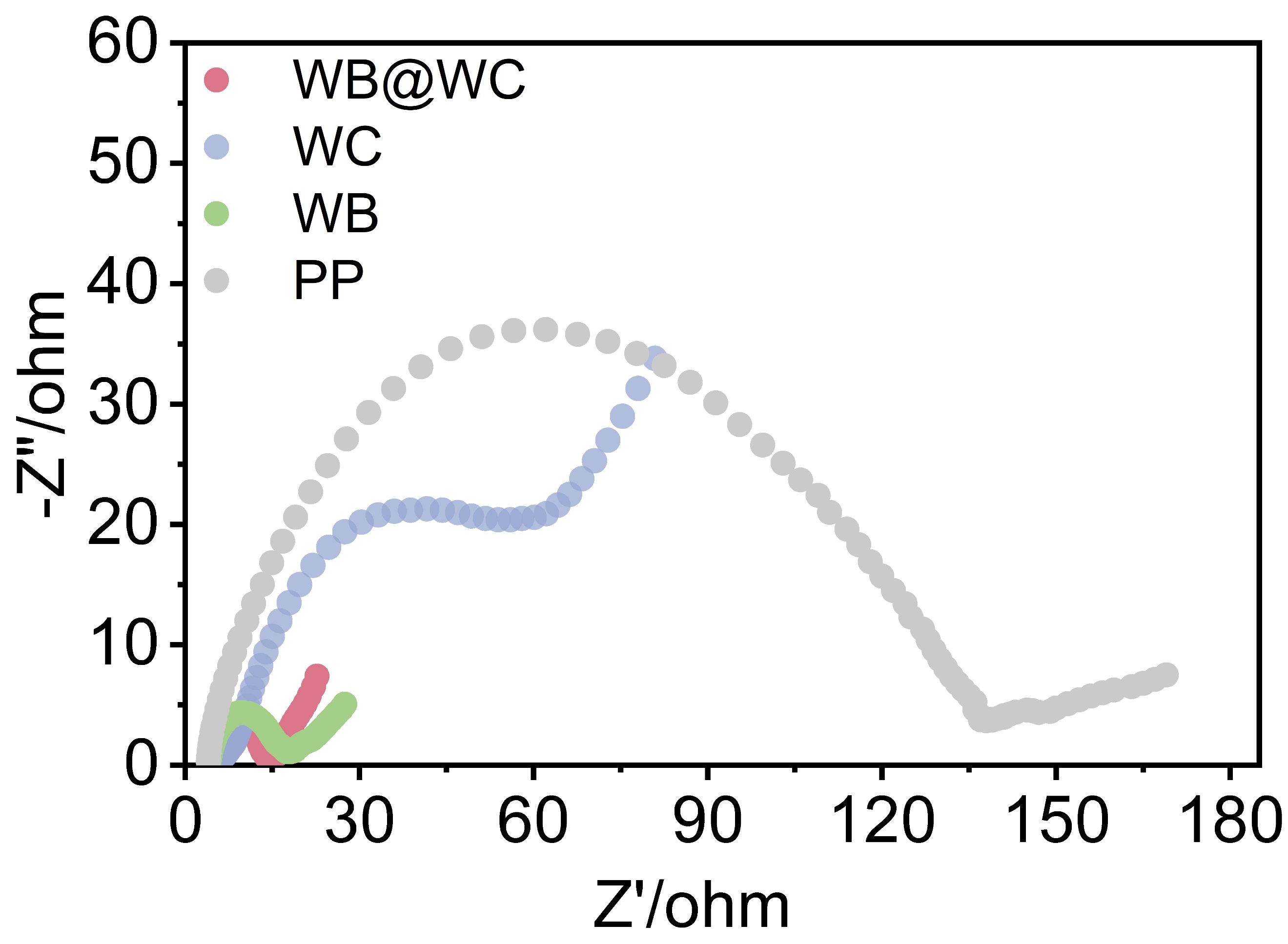


**Fig. S21** EIS of different cells


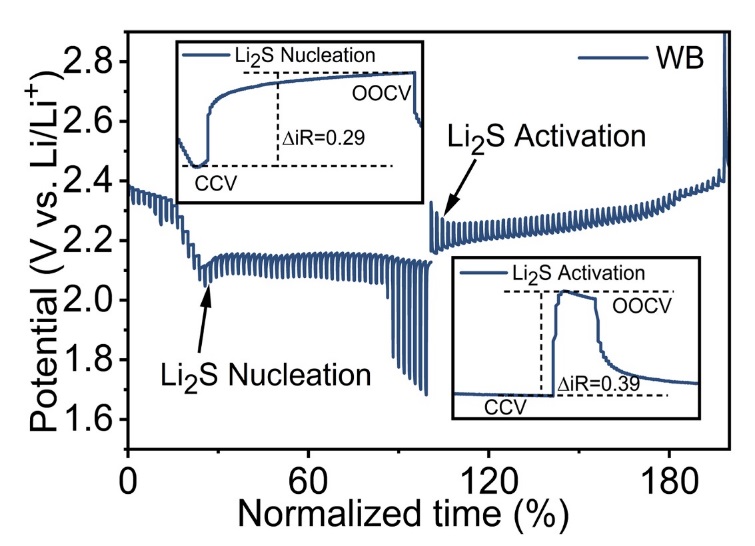


**Fig. S22** GITT voltage profiles of PP based cell at 0.1 C


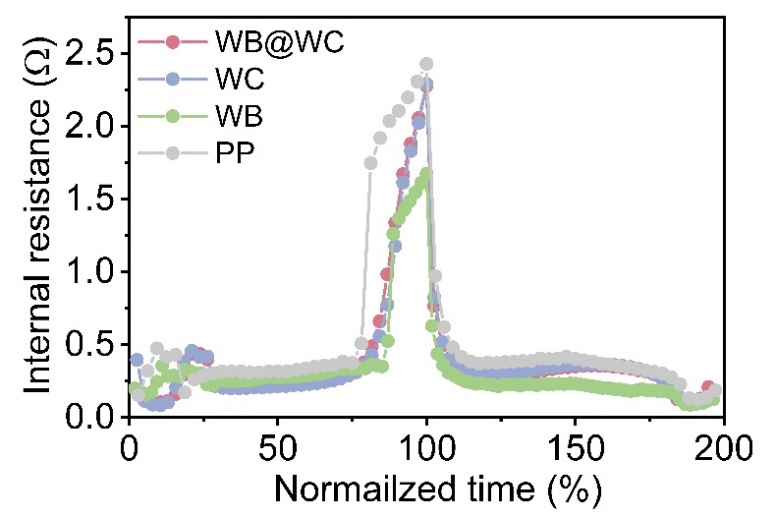


**Fig. S23** Internal resistances relative to the normalized discharge-charge time
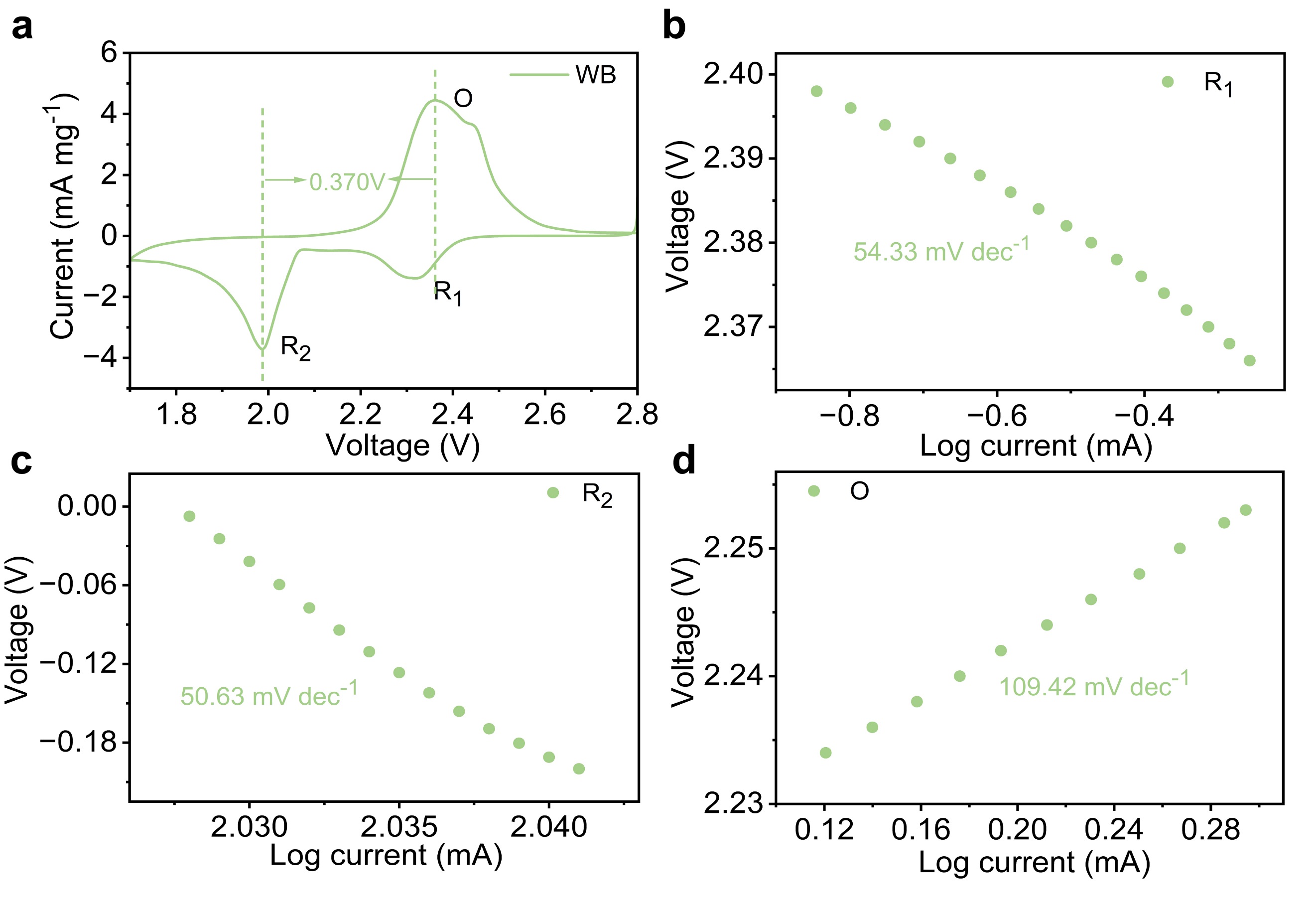


**Fig. S24** **a** CV curve and **b–d** corresponding Tafel fitted slopes of the WB based cathode at 0.1 mV s^−1^


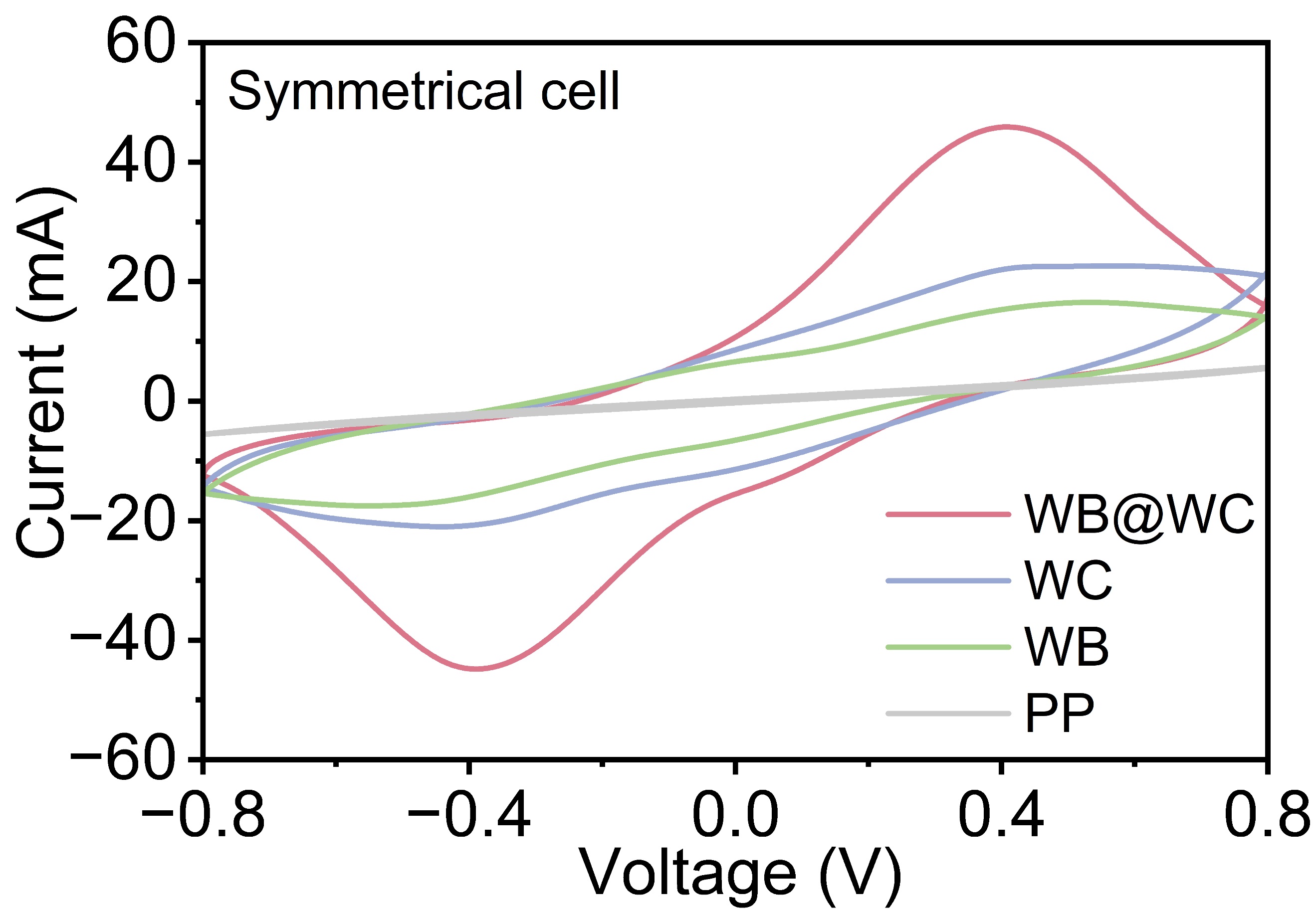


**Fig. S25** Symmetrical cell tests at 50 mV s^−1^


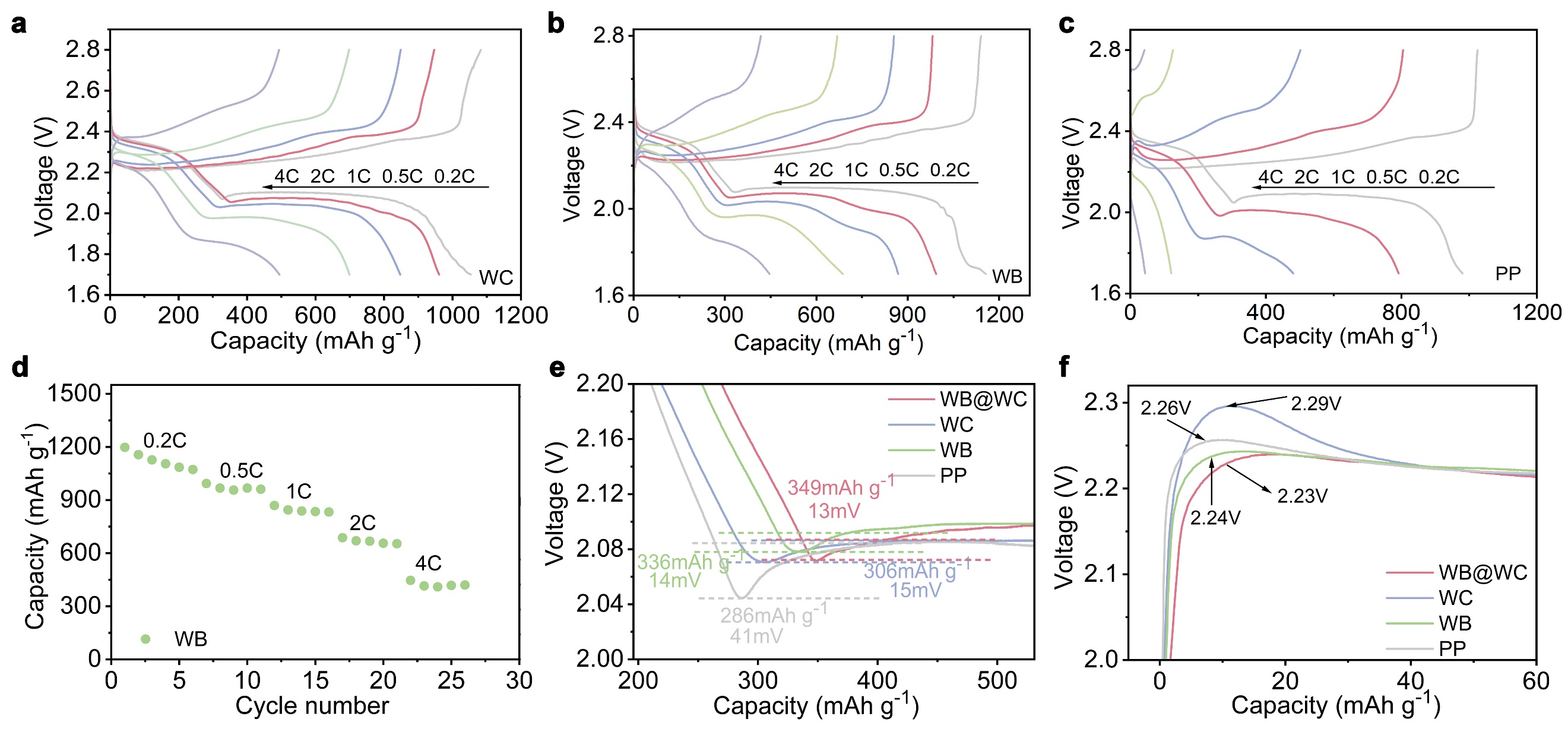


**Fig. S26** **a–c** Voltage profiles of the Li–S cells with different separators at various scan rates. **d** Rate of WB-based cathode. **e**, **f** Partial galvanostatic discharge and charge curves


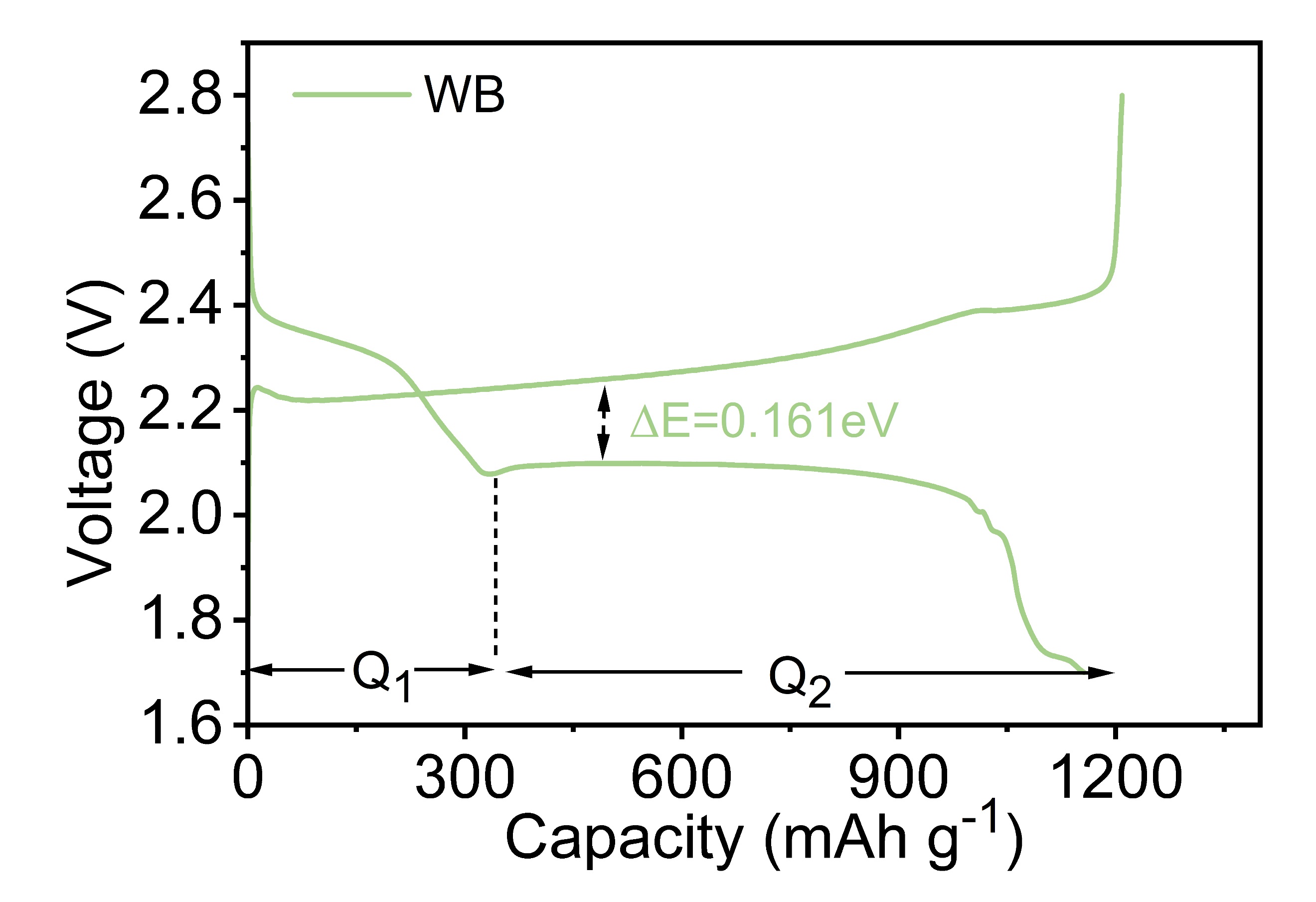


**Fig. S27** Galvanostatic charge-discharge profiles based on WB separator at 0.2 C


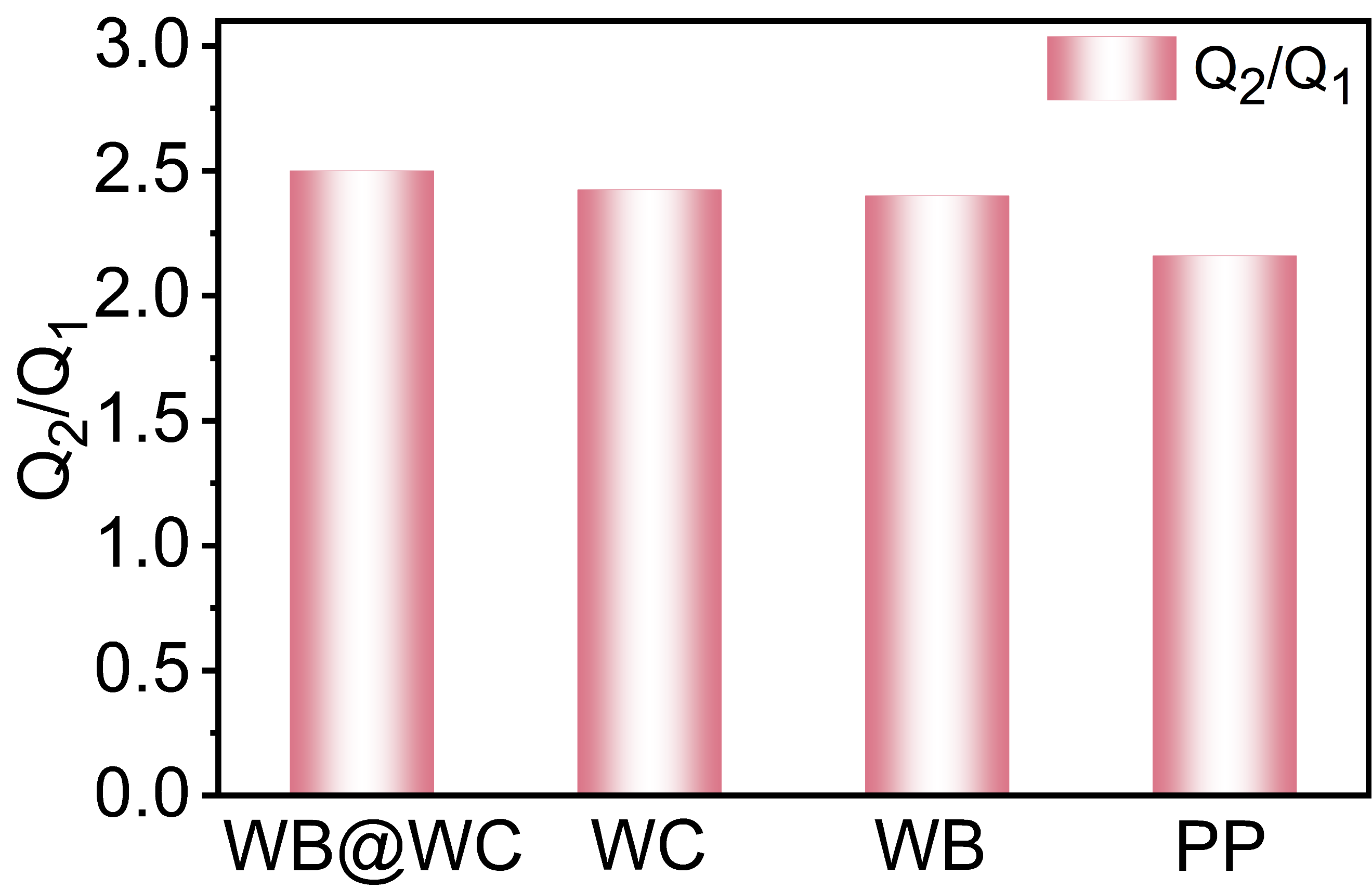


**Fig. S28** The ratio of Q_2_ to Q_1_


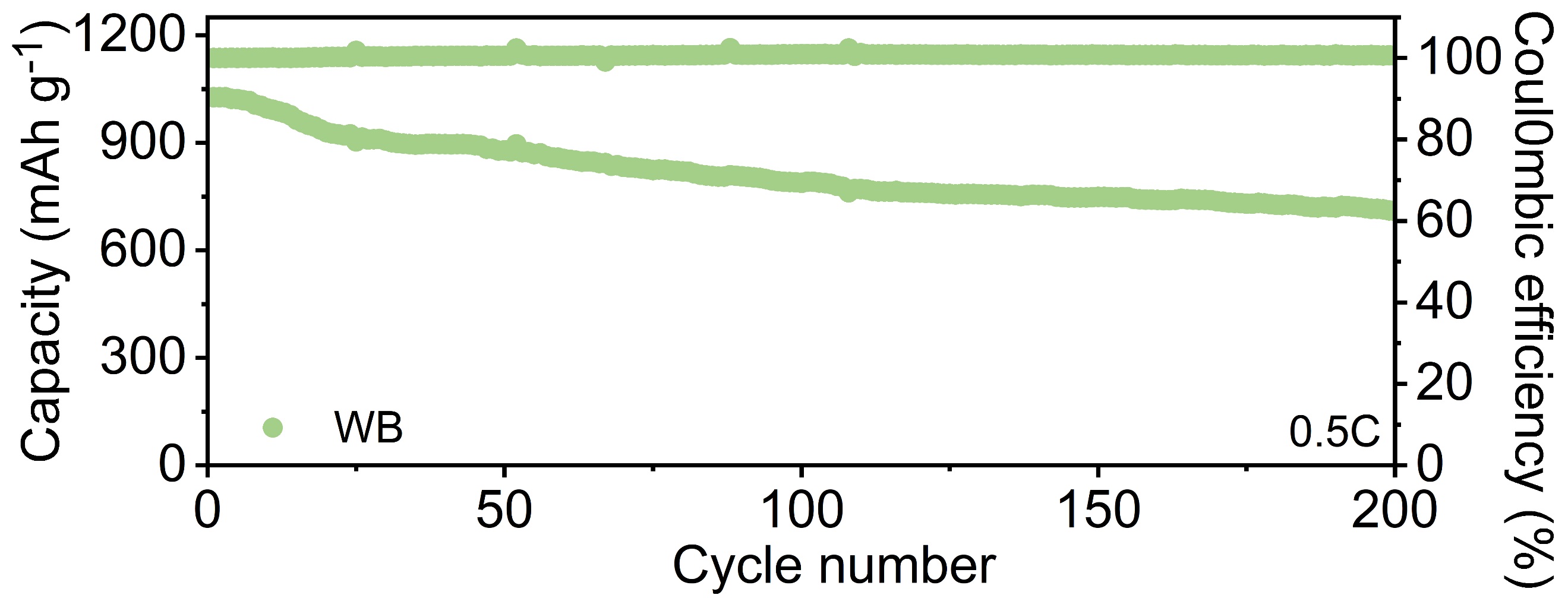


**Fig. S29** Cycling performance of cathode with WB-based separator


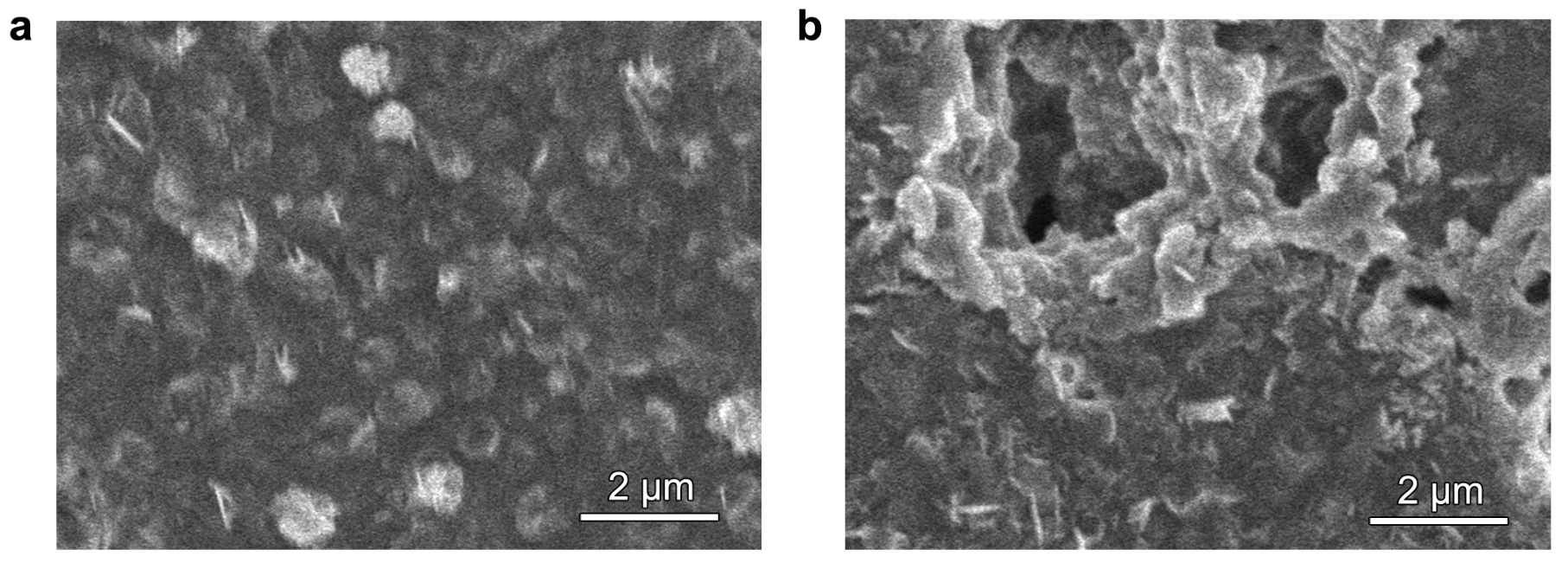


**Fig. S30** Post-mortem SEM analysis of cycled Li anodes: **a** WB@WC modified cell, **b** ordinary cell with pure PP separator


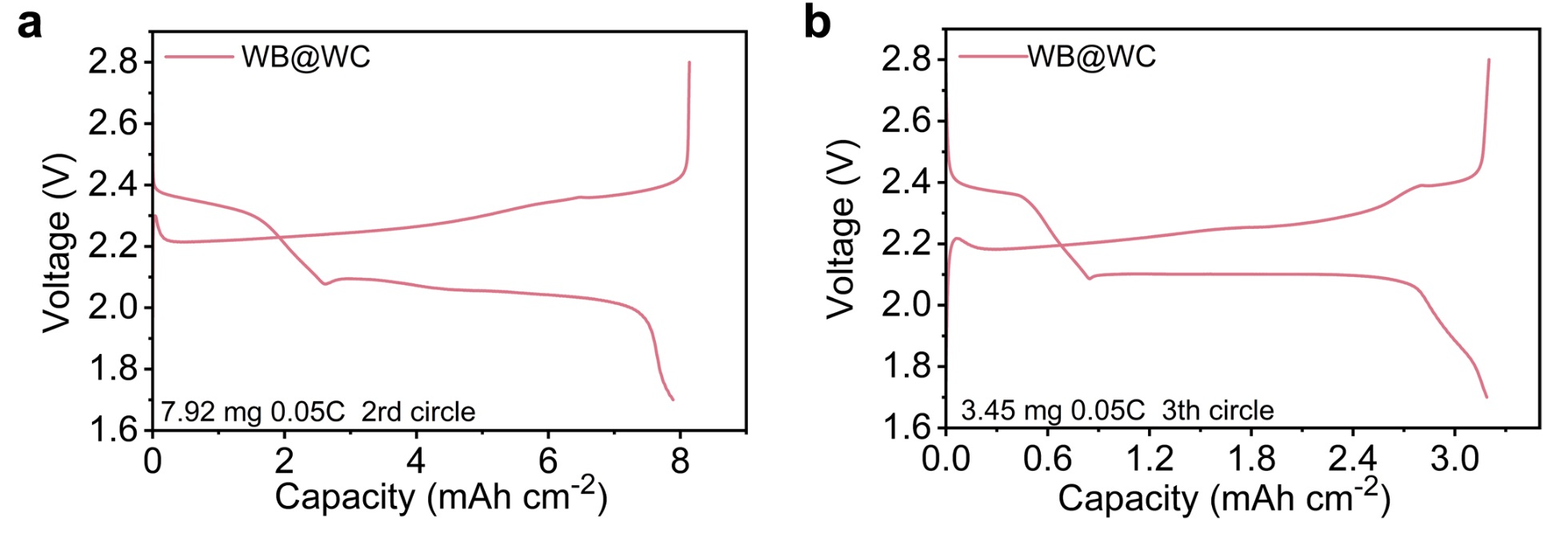


**Fig. S31** Charge-discharge curves under different high loads: **a** 7.92 mg cm^−2^; **b** 3.45 mg cm^−2^


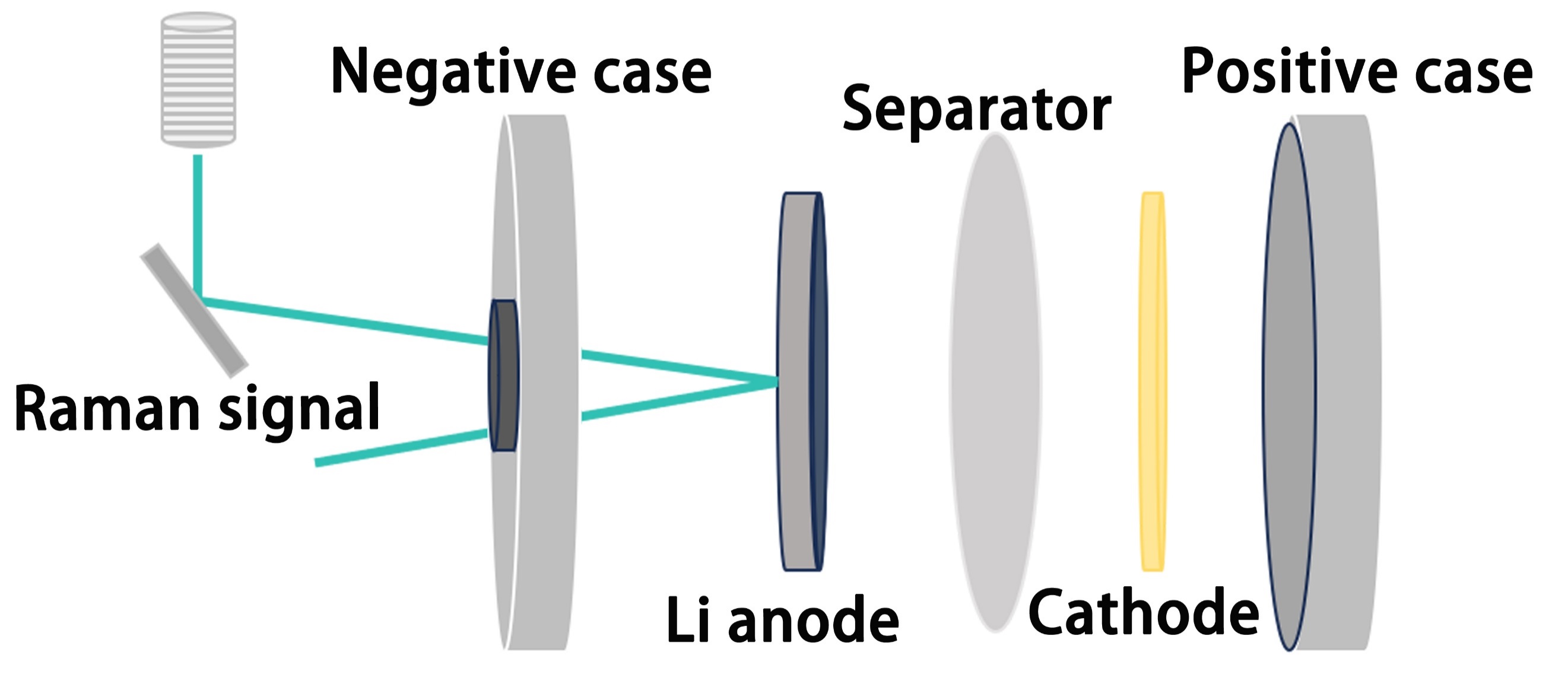


**Fig. S32** Schematic diagram of *in-situ* Raman


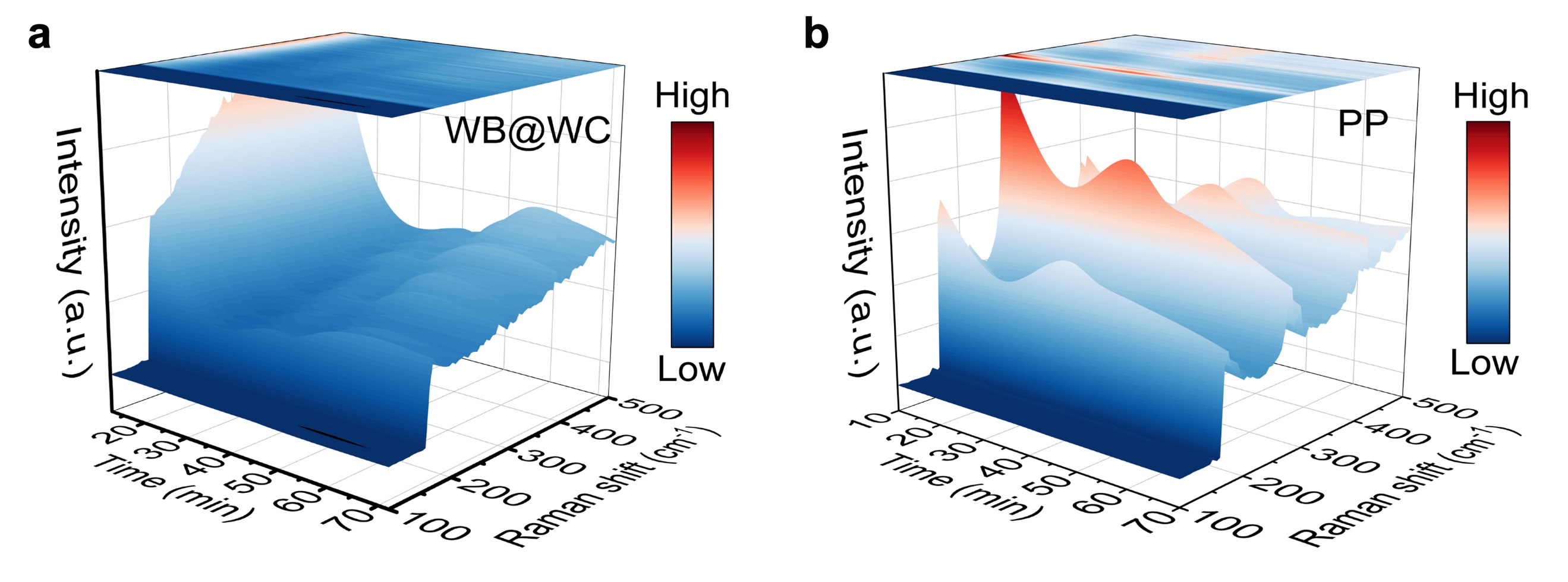


**Fig. S33** 3D *in-situ* time-resolved Raman spectra: **a** WB@WC based cell and **b** PP based cell


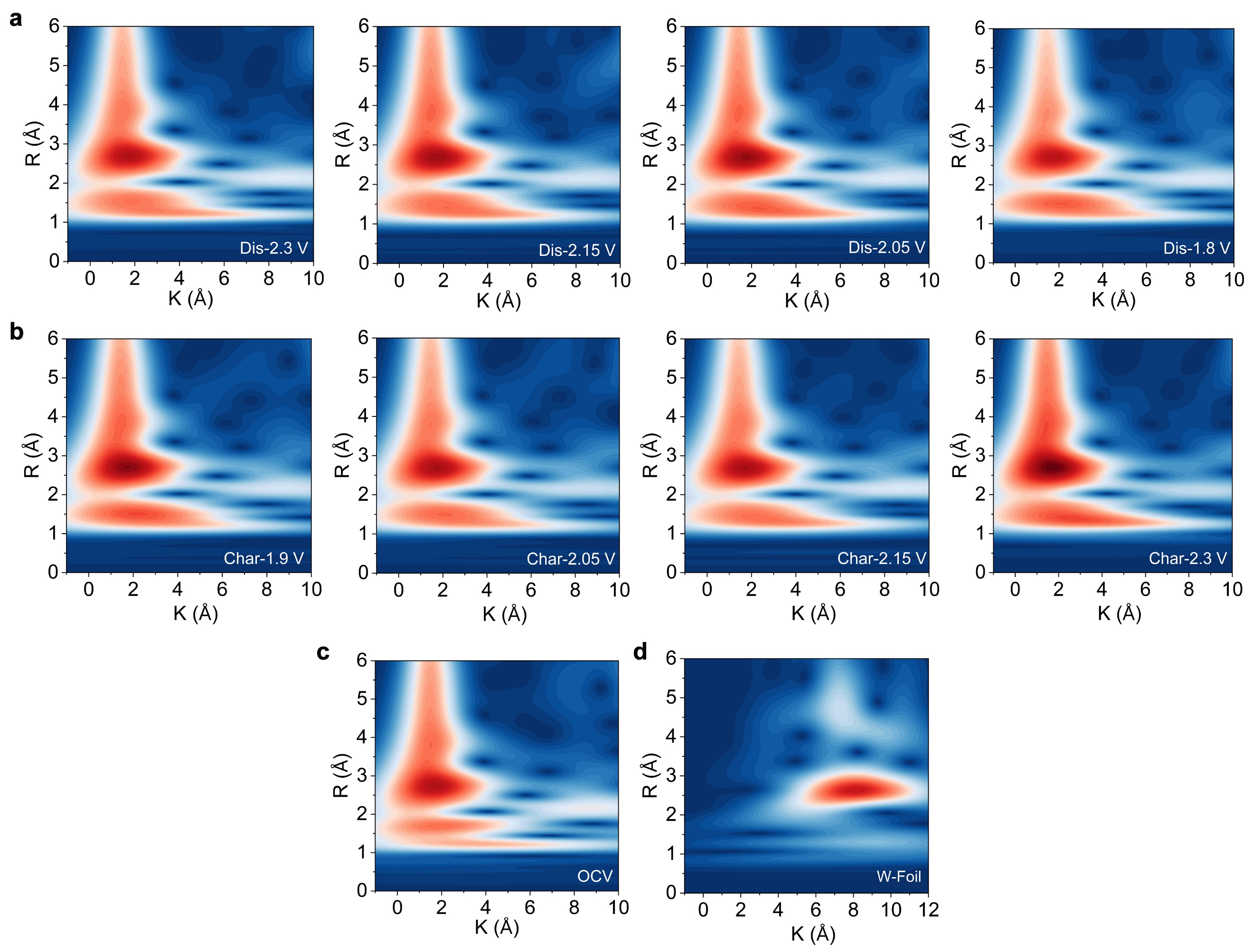


**Fig. S34** *In-situ* WT-XAFS of W L-edge for WB@WC sample

**Table S1** XAFS fitting details

| Sample | Path | C.N | R(Å) | σ^2^*10^-3^(Å^2^) | ΔE(eV) | R factor% |
| --- | --- | --- | --- | --- | --- | --- |
| W foil | W-W1  W-W2 | 8*  6* | 2.74  3.16 | 3.0  3.0 | 6.7 | 1.4% |
| WB | W-B | 8.9 | 2.24 | 3.0 | 9 | 4.6% |
| WB@WC | W-B1  W-C  W-B2 | 1.1  4.7  5.0 | 3.12  2.85  2.22 | 3.0  3.0  3.0 | 4.4 | 2.0% |
| WB@WC-LiPS | W-S  W-C  W-B | 0.8  9.6  8.1 | 2.29  2.71  3.00 | 3.0  3.0  3.0 | -6.7 | 1.8% |
| WC | W-C | 9.1 | 2.06 | 3.0 | -1.8 | 3.8% |
| WS_2_ | W-S | 5.3 | 2.34 | 3.0 | 2.4 | 1.1% |

CN: coordination number; R: distance between absorber and backscatter atoms; σ^2^: Debye-Waller factors; ΔE0: inner potential correction. R-factor: indicative of fitting goodness. The fixed values (*) in the fitting process.

**Table S2** Comparison of the improvement of electrochemical performance by W-based materials in Li_−_S batteries

| W-based nanomaterials | Long cycle capacity | High loading (mg cm^−2^) | Refs. |
| --- | --- | --- | --- |
| rGO@WO_3_ | 57% (500^th^) | 5.0 | [S5] |
| Ni-WS_2_@rGO | 47.8% (500^th^) | 4.5 | [S6] |
| WS_2_-rGO-CNT | 90.7% (100^th^) | 7.68 | [S7] |
| e-WS_2_ | 64.3% (180^th^) | / | [S8] |
| WS_2_/MoS_2_ | 65.5% (300^th^) | 3.0 | [S9] |
| WN-160 | 70.2% (500^th^) | / | [S10] |
| WN-CNS | 80% (500^th^) | 3.0 | [S11] |
| WCNP@HCPC | 80.5% (500^th^) | 5.9 | [S12] |
| 3D WB | 60% (200^th^) | 8.0 | [S13] |
| meso-WC/rGO | 83% (300^th^) | 3.0 | [S14] |
| HC-S + WB_2_/PSS-Li | 75% (500^th^) | 3.8 | [S15] |
| WO_x_/W_2_C | 73.3% (280^th^) | 4.0 | [S16] |
| WS_2_/WO_3_ | 70% (500^th^) | 5.0 | [S17] |
| WO_3-x_-W_3_N_4_ | 54.8% (400^th^) | 11.7 | [S18] |
| WN | 52.4% (500^th^) | 1.4 | [S19] |
| WB@WC | **88% (500^th^)** | **7.92** | **This work** |

**Supplementary References**

1. S. Grimme, Semiempirical GGA-type density functional constructed with a long-range dispersion correction. J. Comput. Chem. **27**(15), 1787–1799 (2006). <https://doi.org/10.1002/jcc.20495>
2. J.P. Perdew, K. Burke, M. Ernzerhof, Generalized gradient approximation made simple. Phys. Rev. Lett. **77**(18), 3865–3868 (1996). <https://doi.org/10.1103/physrevlett.77.3865>
3. S.J. Clark, M.D. Segall, C.J. Pickard, P.J. Hasnip, M.I.J. Probert et al., First principles methods using CASTEP. Z. Für Kristallogr. Cryst. Mater. **220**(5–6), 567–570 (2005). <https://doi.org/10.1524/zkri.220.5.567.65075>
4. Z. Shen, M. Cao, Z. Zhang, J. Pu, C. Zhong et al., Efficient Ni_2_Co_4_P_3_ nanowires catalysts enhance ultrahigh-loading lithium–sulfur conversion in a microreactor-like battery. Adv. Funct. Mater. **30**(3), 1906661 (2020). <https://doi.org/10.1002/adfm.201906661>
5. L. Ni, S. Duan, H. Zhang, J. Gu, G. Zhao et al., A 3D Graphene/WO_3_ nanowire composite with enhanced capture and polysulfides conversion catalysis for high-performance Li–S batteries. Carbon **182**, 335–347 (2021). <https://doi.org/10.1016/j.carbon.2021.05.056>
6. M.A. Al-Tahan, Y. Dong, A.E. Shrshr, X. Liu, R. Zhang et al., Enormous-sulfur-content cathode and excellent electrochemical performance of Li-S battery accouched by surface engineering of Ni-doped WS_2_@rGO nanohybrid as a modified separator. J. Colloid Interface Sci. **609**, 235–248 (2022). <https://doi.org/10.1016/j.jcis.2021.12.035>
7. S. Huang, Y. Wang, J. Hu, Y. Von Lim, D. Kong et al., Mechanism investigation of high-performance Li–polysulfide batteries enabled by tungsten disulfide nanopetals. ACS Nano **12**(9), 9504–9512 (2018). <https://doi.org/10.1021/acsnano.8b04857>
8. A.M. Abraham, S. Ponnurangam, V. Thangadurai, Facet-engineered tungsten disulfide for promoting polysulfide electrocatalysis in lithium–sulfur batteries. Inorg. Chem. **60**(17), 12883–12892 (2021). <https://doi.org/10.1021/acs.inorgchem.1c01241>
9. H. Zhao, H. Wu, J. Wu, J. Li, Y. Wang et al., Preparation of MoS_2_/WS_2_ nanosheets by liquid phase exfoliation with assistance of epigallocatechin gallate and study as an additive for high-performance lithium-sulfur batteries. J. Colloid Interface Sci. **552**, 554–562 (2019). <https://doi.org/10.1016/j.jcis.2019.05.080>
10. Z.-D. Huang, Y. Fang, M. Yang, J. Yang, Y. Wang et al., Sulfur in mesoporous tungsten nitride foam blocks: a rational lithium polysulfide confinement experimental design strategy augmented by theoretical predictions. ACS Appl. Mater. Interfaces **11**(22), 20013–20021 (2019). <https://doi.org/10.1021/acsami.9b04246>
11. H. Liu, H. Shen, R. Li, S. Liu, A. Turak et al., Tungsten-nitride-coated carbon nanospheres as a sulfur host for high-performance lithium-sulfur batteries. ChemElectroChem **6**(7), 2074–2079 (2019). <https://doi.org/10.1002/celc.201900139>
12. J. Zhang, S. Duan, C. You, J. Wang, H. Liu et al., In situ-grown tungsten carbide nanoparticles on nanocarbon as an electrocatalyst to promote the redox reaction kinetics of high-mass loading sulfur cathode for high volumetric performance. J. Mater. Chem. A **8**(42), 22240–22250 (2020). <https://doi.org/10.1039/d0ta07464k>
13. Y. Zhao, C. Liu, C. Zha, J. Li, C. Lyu et al., Tailoring WB morphology enables d-band centers to be highly active for high-performance lithium-sulfur battery. Chin. Chem. Lett. **34**(11), 108189 (2023). <https://doi.org/10.1016/j.cclet.2023.108189>
14. S.-H. Moon, J.-H. Kim, J.-H. Shin, J.-S. Jang, S.-B. Kim et al., High absorption and fast polysulfides conversion of duel functional separator based on mesoporous-WC/rGO composite for lithium-sulfur batteries. J. Alloys Compd. **904**, 164120 (2022). <https://doi.org/10.1016/j.jallcom.2022.164120>
15. T.S. Sahu, V.G. Abhijitha, I. Pal, S. Sau, M. Gautam et al., Regulating polysulfide conversion kinetics using tungsten diboride as additive for high-performance Li-S battery. Small **18**(41), e2203222 (2022). <https://doi.org/10.1002/smll.202203222>
16. B. Wang, R. Fang, K. Chen, S. Huang, R. Niu et al., Heterostructured WO_x_/W_2_C nanocatalyst for Li_2_S oxidation in lithium-sulfur batteries with high-areal-capacity. Small **20**(27), e2310801 (2024). <https://doi.org/10.1002/smll.202310801>
17. B. Zhang, C. Luo, Y. Deng, Z. Huang, G. Zhou et al., Optimized catalytic WS_2_–WO_3_ heterostructure design for accelerated polysulfide conversion in lithium–sulfur batteries. Adv. Energy Mater. **10**(15), 2000091 (2020). <https://doi.org/10.1002/aenm.202000091>
18. D. Zhang, T. Duan, Y. Luo, S. Liu, W. Zhang et al., Oxygen defect-rich WO_3−x_-W_3_N_4_ Mott–Schottky heterojunctions enabling bidirectional catalysis for sulfur cathode. Adv. Funct. Mater. **33**(42), 2306578 (2023). <https://doi.org/10.1002/adfm.202306578>
19. N. Yan, H. Cui, S. You, J. Shi, Y. Weng et al., Tungsten nitride nanotubes as sulfur host material for high performance Li-S batteries. Inorg. Chem. Commun. **140**, 109458 (2022). <https://doi.org/10.1016/j.inoche.2022.109458>
